# Single-cell profiling of peripheral and local immune compartments reveal unique genotype-independent prognostic immune signatures across isocitrate dehydrogenase-stratified glioma

**DOI:** 10.1093/neuonc/noaf206

**Published:** 2025-09-23

**Authors:** Jonathan H Sussman, Anthony R Cillo, Sajeev Kohli, Carly Cardello, Jonathan Patterson, Ebrar Akca, Angelo Angione, David Moon, Katharine Krueger, Ali Ghamari, Emily Xu, Jessica Xu, Antonio C Tarbay, Alex Li, Bhargavi R Budihal, Xiaoran Zhang, Omar Elghawy, Wojciech K Panek, Prajwal Rajappa, Kai Tan, Dario A A Vignali, Tullia C Bruno, Nduka M Amankulor

**Affiliations:** Department of Neurosurgery, Hospital of the University of Pennsylvania, Philadelphia, Pennsylvania, USA; Graduate Group in Genomics and Computational Biology, Perelman School of Medicine, University of Pennsylvania, Philadelphia, Pennsylvania, USA; Department of Immunology, School of Medicine, University of Pittsburgh, Pittsburgh, Pennsylvania, USA; Cancer Immunology and Immunotherapy Program, UPMC Hillman Cancer Center, Pittsburgh, Pennsylvania, USA; Center for Systems Immunology, University of Pittsburgh, Pittsburgh, Pennsylvania, USA; Department of Neurosurgery, Hospital of the University of Pennsylvania, Philadelphia, Pennsylvania, USA; Graduate, Group in Cellular and Molecular Biology, Perelman School of Medicine, University of Pennsylvania, Philadelphia, Pennsylvania, USA; Department of Immunology, School of Medicine, University of Pittsburgh, Pittsburgh, Pennsylvania, USA; Tumor Microenvironment Center, UPMC Hillman Cancer Center, Pittsburgh, Pennsylvania, USA; Department of Neurosurgery, Hospital of the University of Pennsylvania, Philadelphia, Pennsylvania, USA; Department of Neurosurgery, Hospital of the University of Pennsylvania, Philadelphia, Pennsylvania, USA; Department of Neurosurgery, Hospital of the University of Pennsylvania, Philadelphia, Pennsylvania, USA; Department of Neurosurgery, Hospital of the University of Pennsylvania, Philadelphia, Pennsylvania, USA; Department of Neurosurgery, Hospital of the University of Pennsylvania, Philadelphia, Pennsylvania, USA; Department of Neurosurgery, Hospital of the University of Pennsylvania, Philadelphia, Pennsylvania, USA; Department of Neurosurgery, Hospital of the University of Pennsylvania, Philadelphia, Pennsylvania, USA; Graduate Group in Genomics and Computational Biology, Perelman School of Medicine, University of Pennsylvania, Philadelphia, Pennsylvania, USA; Center for Systems Immunology, University of Pittsburgh, Pittsburgh, Pennsylvania, USA; Graduate Group in Genomics and Computational Biology, Perelman School of Medicine, University of Pennsylvania, Philadelphia, Pennsylvania, USA; Center for Systems Immunology, University of Pittsburgh, Pittsburgh, Pennsylvania, USA; BGS Global Institute of Medical Sciences, Bengaluru, India; Department of Immunology, School of Medicine, University of Pittsburgh, Pittsburgh, Pennsylvania, USA; Tumor Microenvironment Center, UPMC Hillman Cancer Center, Pittsburgh, Pennsylvania, USA; Department of Medicine, Perelman School of Medicine, University of Pennsylvania, Philadelphia, Pennsylvania, USA; Department of Clinical Sciences and Advanced Medicine, School of Veterinary Medicine, University of Pennsylvania, Philadelphia, Pennsylvania, USA; Department of Pediatrics, The Ohio State Wexner Medical Center, Columbus, Ohio, USA; Department of Neurological Surgery, The Ohio State Wexner Medical Center, Columbus, Ohio, USA; Department of Pediatrics, Children’s Hospital of Philadelphia, Philadelphia, PA, USA; Department of Immunology, School of Medicine, University of Pittsburgh, Pittsburgh, Pennsylvania, USA; Tumor Microenvironment Center, UPMC Hillman Cancer Center, Pittsburgh, Pennsylvania, USA; Cancer Immunology and Immunotherapy Program, UPMC Hillman Cancer Center, Pittsburgh, Pennsylvania, USA; Department of Immunology, School of Medicine, University of Pittsburgh, Pittsburgh, Pennsylvania, USA; Tumor Microenvironment Center, UPMC Hillman Cancer Center, Pittsburgh, Pennsylvania, USA; Cancer Immunology and Immunotherapy Program, UPMC Hillman Cancer Center, Pittsburgh, Pennsylvania, USA; Department of Neurosurgery, Hospital of the University of Pennsylvania, Philadelphia, Pennsylvania, USA; Penn Brain Tumor Center, Abramson Cancer Center, Perelman School of Medicine, University of Pennsylvania, Philadelphia, PA, USA

**Keywords:** circulating immune cells, IDH Mutant glioma, immunotherapy, single-cell sequencing, tumor immune microenvironment

## Abstract

**Background:**

Solid tumor immune suppression requires cooperation of tumor cells, local immune cells, peripheral circulating immune cells, and evolution of immune cell trajectories between peripheral and local environments. This study addresses a significant knowledge gap by characterizing peripheral and local immune environments in isocitrate dehydrogenase (IDH) Mutant (IDH-Mut) and IDH wildtype (IDH-WT) gliomas and defines novel immunological states with prognostic relevance across the glioma landscape.

**Methods:**

We analyzed local and peripheral immune phenotypes in a cohort of 18 (6 IDH-Mut and 12 IDH-WT) gliomas with distinct genetic characteristics using paired human tumor and peripheral blood mononuclear cells (PBMCs) with single-cell RNA-sequencing (scRNA-seq).

**Results:**

Our analyses revealed unique intratumoral and peripheral immune cellular ontogenies, including naïve CD4^+^ T cell enrichment in the IDH-Mut peripheral immune compartment, monocyte enrichment in IDH-WT glioma PBMCs, and emergence of a unique population of GZMH^+^ CD8^+^ T cells preferentially in the IDH-Mut microenvironment. Additionally, we found upregulation of TNF-α signaling and inflammatory response pathways in IDH-Mut-­glioma-associated peripheral lymphoid cells versus IDH-WT tumors and identified a novel population of microglia-like cells in the peripheral blood of glioma patients with complement-interfacing characteristics. Applying intratumoral transcriptomic deconvolution via The Cancer Genome Atlas revealed genotype-independent, prognostic immune signatures across the malignant glioma landscape.

**Conclusions:**

This study reveals variable expression of immune phenotypes in adult gliomas stratified by IDH status and characterizes immune compartment and genotype-dependent differences in the immunologic glioma landscape. These genotype-dependent, tumor and circulating immune ontogenies should guide future diagnostic and immunotherapeutic considerations in malignant glioma.

## Graphical Abstract

**Graphical Abstract:**
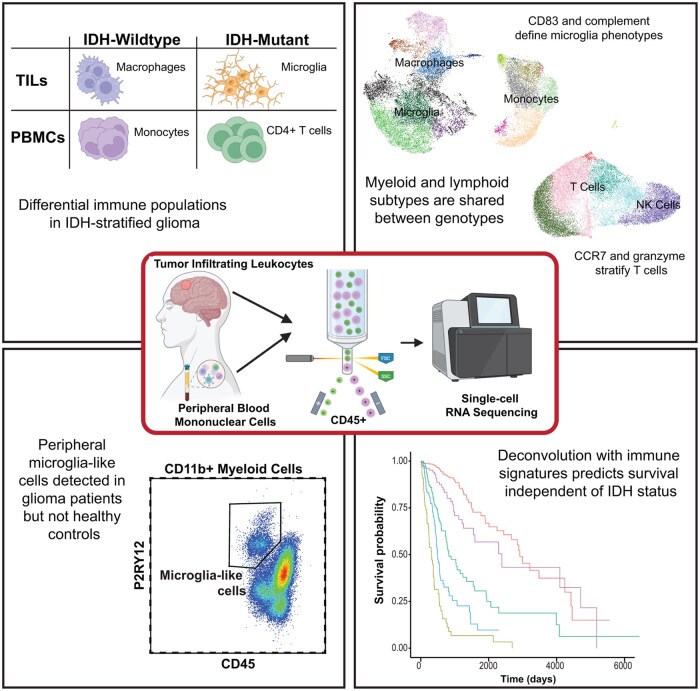

Key Points

1. IDH mutations are associated with distinct peripheral immune landscapes.
2. Microglia-like cells are present in the peripheral blood of glioma patients.
3. Immune signatures predict overall survival in an IDH-independent manner.

Importance of the Study

This study substantially advances our understanding of immune dynamics in IDH-mutant and IDH-wildtype gliomas by exploring matched local tumor and circulating immune ontogenies using single-cell RNA sequencing. Building on prior research into the glioma immune microenvironment, the integrative analysis presented herein uncovers distinct IDH-dependent immune profiles within intratumoral and circulating immune cells. Furthermore, the current study demonstrates and validates the existence of circulating, functional microglia-like cells capable of modulating lymphocyte function. By integrating bulk RNA sequencing data from The Cancer Genome Atlas, we demonstrate novel prognostic immunologic signatures that independently predict overall survival. Collectively, this work amplifies our current understanding of global immune responses to IDH-wildtype and IDH-mutant glioma, provides a framework for the development of novel immunotherapies and diagnostic biomarkers, and suggests a potential role for natural immunity in glioma.

Diffuse gliomas are incurable malignancies of the central nervous system. Glioblastoma (GBM), its most aggressive form, is associated with a median overall survival (OS) of 14 months.^1^ While diffuse low-grade gliomas (DLGG) characterized by recurrent IDH mutations are associated with better prognosis (overall survival ∼5-20 years), they inevitably progress to high-grade glioma and are uniformly fatal,^2^ with younger age at mortality than IDH-wildtype (WT) glioma. Importantly, gliomas are highly heterogeneous, with variable histologic architecture, tumor microenvironments, gene expression profiles, and clinical outcomes.^1^ Mutations in isocitrate dehydrogenase (*IDH*) define an important subtype of glioma and are found in the majority of DLGG. Mechanisms of oncogenesis in IDH-mutant (Mut) gliomas are fundamentally different from those of IDH-WT tumors,^3^^,^^4^ including oncometabolite transformation and the induction of unique methylation signatures.^5^ The tumor immune microenvironment (TIME), an important driver of oncogenic transformation, is also radically different across glioma genotypes.^6-8^ For instance, early studies from our group established that IDH mutations induce immunologically cold local TIME signatures punctuated by dramatic loss of lymphocytes and the infiltration of suppressive myeloid phenotypes capable of promoting immune evasion and tumor progression.^6^ A practical consequence of this finding is that the immunosuppressive cellular states induced in part by the oncometabolite 2-hydroxyglutarate likely hinder the efficacy of immune checkpoint blockade in IDH-Mut glioma given the absence of lymphocytes capable of effector function.^9^^,^^10^

Beyond the TIME, neoplasia induces systemic changes in the peripheral immune system that can serve as prognostic and diagnostic biomarkers in multiple tumor types.^11-14^ Knowledge of the immunologic landscape of solid tumor malignancies may enhance the development of cellular immunotherapy approaches, including checkpoint inhibition using rational tumor genotype-driven paradigms.^15^^,^^16^ Foundational glioma immunology studies have demonstrated that IDH-WT glioma induces striking changes in peripheral blood immune populations. These studies demonstrate that peripheral CD8^+^ T cells^17^ as well as myeloid-derived suppressor cells are associated with poor prognosis, suggesting a failure of antigen-specific T-cells in the TIME.^18^^,^^19^ We have demonstrated *in vivo* that IDH-Mut glioma induces systemic immunosuppression through the release of small extracellular vesicles into the peripheral circulation.^20^

The breadth and resolution of single-cell RNA-sequencing (scRNA-seq) have enhanced characterization of the TIME in glioma and have transformed our understanding of peripheral and local immune response during glioma progression and therapeutic resistance.^7-9^^,^^21-31^ Nevertheless, the potential mechanistic and prognostic consequences of distinct circulating immune cells in IDH-WT and IDH-Mut glioma are insufficiently characterized at the single-cell level. In this study, we performed scRNA-seq on intratumoral and peripheral CD45^+^ immune cells across 12 IDH-WT and 6 IDH-Mut glioma patients. Through an integrated analysis, we identified significant differences in the immune composition of the tumor microenvironment and peripheral blood between IDH-WT and IDH-Mut glioma patients, and we identified microglia-like cells in the peripheral blood of glioma patients. Subsequently, we uncovered distinct prognostic immune signatures that predict overall survival in a genotype-independent fashion in both IDH-WT and IDH-Mut glioma.

## Methods

### Patient Cohort for Single-Cell RNA-Seq Profiling

Consecutive patients undergoing resection as treatment for glioma were enrolled, after written informed consent was obtained in accordance with the Declaration of Helsinki and Institutional Review Board Approval from the University of Pittsburgh Medical Center under UPMC tissue collection protocol 99-069. Patients had primary (non-recurrent) disease and did not receive any glioma therapy before tissue collection. We required that >70% extent of resection be achieved, and ∼4 g of grossly viable non-necrotic tissue could be obtained, confirmed by histopathology, to ensure broad representation of tumor regions. There were no other exclusion criteria. The Chromium Single Cell 3′ Reagent Kit v2 or 5′ Reagent Kit v1 (10× Genomics) were used to generate libraries for scRNA-seq. Data were analyzed using Seurat v4 using the standard workflows for data integration and clustering. Detailed materials and methods are available in Supplementary Materials.

## Results

### Single-Cell Profiling of Glioma-Infiltrating and Peripheral Blood Immune Cells Reveals Disparate Genotype-Predominant Myeloid and Lymphoid Ontogenies

**Figure 1.**
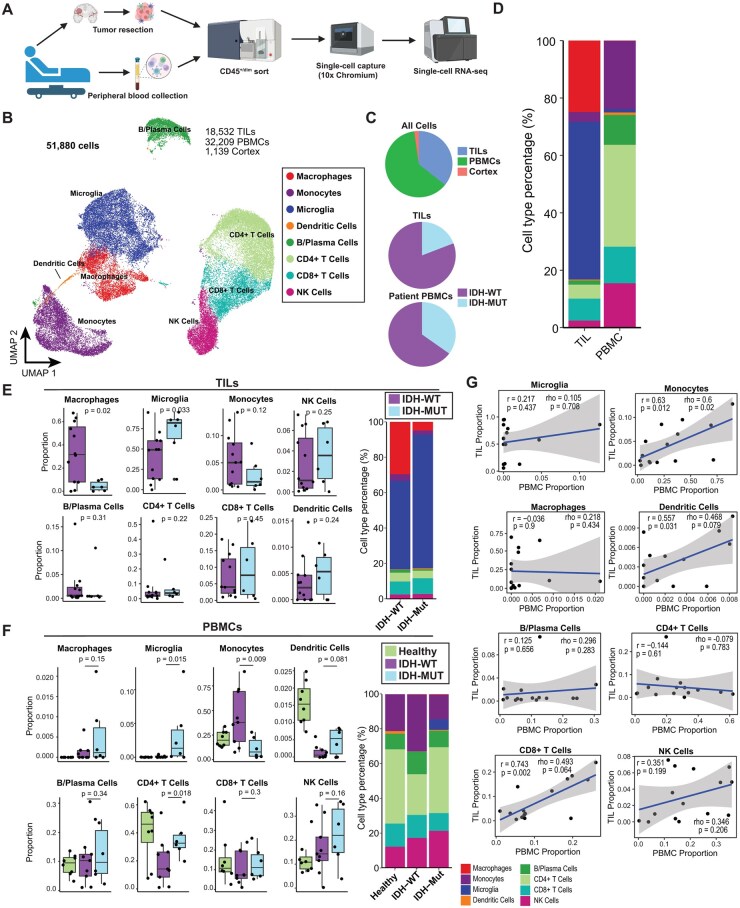
Single-cell transcriptomic profiling of intratumoral and peripheral immune response to glioma. (A) Schematic of experimental workflow. Dissociated tumor tissue and peripheral blood underwent fluorescence-activated cell sorting to isolate CD45^+^ immune cells, followed by scRNA-seq profiling and analysis using the 10× Genomics platform. Created in BioRender. Tan, K. (2025) (https://BioRender.com/1cg96ei). (B) Uniform Manifold Approximation and Projection projection of all cells after filtering for low-quality cells and neuroglial cells (51,880 cells), annotated by coarse cell type. (C) Composition of scRNA-seq dataset highlighting contribution of cells from different tissue types and isocitrate dehydrogenase (IDH) mutation status (D) Barplot showing distribution of cell types characterized in TILs and peripheral blood mononuclear cells (PBMCs), respectively.(E-F) Cell type composition of (E) tumor-infiltrating leukocytes (TILs) and (F) PBMCs comparing IDH-WT and IDH-Mut gliomas. Left, boxplots showing the percentage of each coarse cell type across samples. Samples with at least 25 cells were included (TILs, *n* = 12 IDH-WT, 6 IDH-Mut; PBMCs, *n* = 9 IDH-WT, 6 IDH-Mut). Significance assessed using a one-sided Wilcoxon rank-sum test. Right, barplots showing differences in overall cell type distribution between IDH-WT and IDH-Mut glioma patients. Correlations between cell type proportions in PBMCs and TILs for each cell type population with Pearson’s correlation coefficient (*r*) and Spearman’s correlation coefficient (rho), along with respective *P* values.

To characterize the global immune response to glioma pathogenesis, we profiled CD45^+^ sorted tumor-infiltrating leukocytes (TILs) from 18 treatment-naïve patients with grade II-IV glioma, of which 12 were IDH-WT and 6 were IDH-Mut by histological diagnosis. Microglia are CD45^mid^ and express lower levels of CD45 than peripheral leukocytes,^32^ so we used a broad CD45 gate in order to capture low CD45-expressing immune cells. We also profiled matched peripheral blood mononuclear cells (PBMCs) from 15/18 patients following intubation and, importantly, before administration of pre-operative steroids. (Figure 1A). Our cohort possessed a spectrum of secondary mutations in genes such as EGFR, ATRX, p53, CDKN2A, as well as 1p/19q codeletion and MGMT promoter methylation status (Table S1). We additionally included PBMCs from 8 healthy donors and profiled non-neoplastic cortical tissue isolated from normal adjacent cortical/white matter tissue (en route to resection) in 2 of the glioma patients from the study population, where the cortex and juxtacortical white matter were radiographically normal. In total, we analyzed over 51 880 cells after data filtering, with an average of 3456 unique UMIs and 1090 genes detected per cell. Sequencing chemistry-induced batch effect was computationally corrected, yielding minimal technology-associated batch effect across patients (Figure S1A-C).

We first identified core categories of myeloid cells (microglia, bone marrow-derived macrophages, dendritic cells, and monocytes), and lymphoid cells (CD4^+^ T cells, CD8^+^ T cells, B/plasma cells, and NK cells) via marker gene expression (Figure 1B-D, Figure S1D). Examination of intratumoral immune cell frequencies (TIL compartment) demonstrated a predominance of microglia in IDH-Mut glioma, while IDH-WT gliomas exhibited a higher proportion of tissue macrophage-like cells, consistent with prior reports of malignant glioma^28^^,^^33^ (Figure 1E, Figure S1E). The proportion of T cells and NK cells was comparable between IDH-Mut and IDH-WT glioma (Figure 1E). Notably, CD4^+^ T cells were enriched in the peripheral blood of IDH-Mut patients compared to IDH-WT patients, while IDH-WT patients had a significantly higher frequency of monocyte populations (Figure 1F). Perhaps because we utilized the same permissive CD45 gating for both TILs and PBMCs, we identified a population of cells that transcriptionally clustered alongside brain-resident microglia in the peripheral blood of glioma patients. Remarkably, no microglia-like cells were present in healthy donor samples (Figure 1F). We additionally examined the correlation between immune cell type composition in the TIME and peripheral blood in the 15 glioma patients with temporally-paired scRNA-seq profiling of PBMCs and TILs. In general, CD8^+^ T cells and monocytes displayed a high correlation between PBMCs and TILs regardless of genotype (Figure 1G). Indeed, our broad cell-type phenotypic analysis suggested that lymphocytes correlate most robustly from peripheral to local tumor environments and suggest that lymphocyte-focused circulating immune analysis can reveal insights into the state of the tumor microenvironment.

### IDH-Mut Glioma is Characterized by CD83-Expressing Microglia

**Figure 2.**
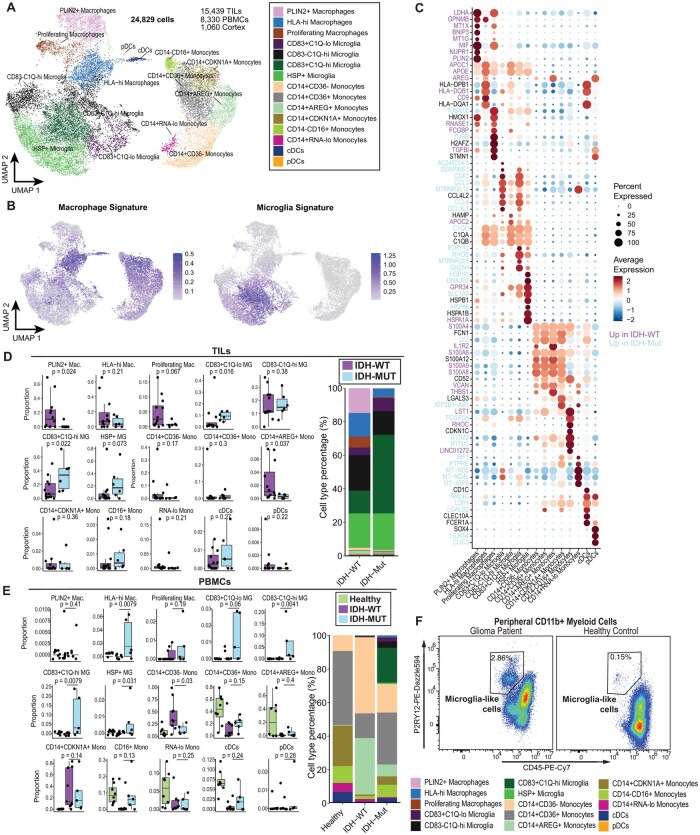
CD83-expressing microglia-like cells are identified in the peripheral blood of glioma patients. (A) UMAP projection showing 15 cell populations of myeloid cells consisting of microglia, macrophages, monocytes, and dendritic cells (24 829 cells). (B) Uniform Manifold Approximation and Projection (UMAP) projection of dataset colored by gene signature scores previously established to distinguish bone marrow-derived macrophages (left) and microglia (right).^29^ Signature score expression is truncated at the 1st and 99th percentile for visualization. (C) Dot plot showing expression level and frequency of cells of the top 8 differentially expressed genes for each myeloid cell type. Dot sizes indicate the percentage of cells in each cluster expressing the genes, and colors indicate the relative average normalized expression level. Gene names are colored if they are differentially expressed (*P*-adjusted < .05) across all cells derived from IDH-WT versus IDH-Mut patients. (D)-(E) Composition of myeloid subtypes in (D) tumor-infiltrating leukocytes (TILs) and (E) peripheral blood mononuclear cells comparing IDH-WT and IDH-Mut gliomas. Left, boxplots showing the percentage of subtype across samples as a fraction of all myeloid cells. Samples with at least 25 cells were included (TILs, *n* = 12 IDH-WT, 6 IDH-Mut; PBMCs, *n* = 9 IDH-WT, 5 IDH-Mut). Significance assessed using a one-sided Wilcoxon rank-sum test. Right, barplots showing differences in overall cell type distribution between IDH-WT and IDH-Mut glioma patients. MG, microglia. (F) Representative example of a population of microglia-like CD45^mid^ P2RY12^+^ cells in the peripheral blood of a glioma patient and healthy control, within the population of CD11b^+^ myeloid cells.

Next, we sought to determine the genotype-specific identities of tumor-associated myeloid cells from the periphery to the local TIME. Given the substantial scRNA-seq intra-compartment heterogeneity in myeloid markers (across both IDH-Mut and IDH-WT gliomas, Figure S1D), we re-clustered the myeloid cells (microglia, macrophages, and monocytes) at a higher resolution. We captured 15 unique myeloid clusters, including 3 macrophage clusters, 4 microglia clusters, 4 monocyte clusters, and 2 clusters of conventional and plasmacytoid dendritic cells, respectively (Figure 2A). Microglia were annotated using a well-defined gene signature stratifying microglia and bone marrow-derived macrophages.^29^ Analysis of myeloid single-cell characteristics across both genotypes revealed a phenotypic spectrum of macrophage and microglial signatures with predominance of microglial signatures in IDH-Mut TILs (Figure 2B**)**. Macrophages stratified into a population with high expression of MHC Class II genes (eg *HLA-DQA1, HLA-DQB1*) and a population with marked overexpression of *PLIN2* (Figure 2C, Table S2). *PLIN2^+^* macrophages expressed high levels of pro-angiogenic (*MIF, SPP1, FN1*)^34^ and metabolic (*HK2, LDHA, ALDOA*) genes. Microglia, in contrast to macrophages, exhibited strata based on combinatorial expression of *CD83* and complement genes (*C1QA, C1QB, C1QC*). *CD83^+^C1Q^hi^* microglia displayed robust expression of multiple chemokines (*CCL4, CCL3, CXCL16*) as well as genes known to drive glioma immunosuppression, including *TREM2*^35^ and *CSF1R*^36^ (Figure 2C). Additional analysis with SCENIC inferred transcriptional induction of TCF7 and MLXIPL in *CD83^+^C1Q^hi^* microglia (Figure 2A and B, Table S3). *CD83*^–^*C1Q^hi^* microglia differentially expressed genes characteristic of lipid-associated TAMs (eg *APOC1, APOE*)^37^ and *CD83^+^C1Q^lo^* microglia differentially expressed similar chemokines to *CD83^+^C1Q^hi^* microglia without marked expression of *CD83* (Figure 2E). A final microglia population was characterized by a broad expression of heat shock protein genes and was termed *HSP^+^* microglia. Notably, both *CD83^+^C1Q^hi^* and *CD83^+^C1Q^lo^* microglia were enriched in the IDH-Mut myeloid compartment compared to IDH-WT glioma. Conversely, *PLIN2^+^* macrophages were more frequent in IDH-WT glioma (Figure 2D). Since IDH-Mut gliomas harbor uniquely immunosuppressive microglial phenotypes,^6^ we postulate that the IDH-enriched microglial subsets revealed in this study represents a key genotype-specific immunosuppressive driver in the IDH-Mut TIME.

### Circulating Myeloid Cells in Glioma Are Genotype-Specific and Feature a Microglia-like Subset

To identify key genotype-dependent populations across circulating immune cells in glioma, we explored the peripheral myeloid cell profiles. Not surprisingly, we first observed that monocytes were the predominant myeloid cell type in the peripheral blood across all glioma genotypes (Figure 1F). We identified several distinct populations of classical (*CD14^+^CD16*^–^) monocytes and one population of nonclassical monocytes (*CD14*^–^*CD16^+^*) with expression of interferon response genes (ie *IFITM2, IFITM3*) (Figure 2C, Table S2) and differentially featuring activation of the HES4 transcriptional regulon (Figure S2C-D). Expression of monocyte-related genes (ie *S100A8, LYZ, FCN1, FCGR*) was high across the monocyte clusters; however, transcriptionally distinct monocyte subsets emerged within the larger classical and nonclassical umbrella.

Unsupervised clustering of the monocytes across the dataset revealed three unique clusters: *CD36^hi^* monocytes, *CDKN1A-*expressing monocytes, and a cluster marked by high expression of non-canonical EGFR ligands *AREG* and *EREG*^23^ as well as enrichment of the BCL6 regulon (Figure 2C, Figures S2A-B, Table S3). Single-cell sequencing also captured genotype-specific monocyte states largely restricted to IDH-Mut or IDH-WT gliomas. For instance, *CD14^+^CD36^–^* monocytes were more frequent in the PBMCs of IDH-WT patients, while nonclassical *CD16^+^* monocytes were more frequent in IDH-Mut PBMCs (Figure 2E).

We observed a small number of PBMCs in most patients that clustered closely with brain-resident microglia and were therefore annotated as such (Figure 1A-D**)**. Most circulating microglia-like cells were predominantly seen in three IDH-Mut glioma patients from our total cohort. We confirmed that PBMCs annotated as “microglia” indeed expressed high levels of the canonical microglial markers *P2RY12* and *TMEM119* comparable to intratumoral microglia and confirmed the presence of circulating microglia-like cells in glioma patients relative to healthy controls (Figure 1F, ­Figure S3A-B). The unexpected finding of circulating blood microglia-like cells prompted further exploration using RNA velocity analysis, which infers differentiation trajectories through the ratio of spliced to unspliced RNA in each cell. This analysis revealed that *HLA^hi^* macrophages exist in the middle of a differentiation hierarchy bifurcating into 2 distinct paths: (1) toward *PLIN2^+^* macrophages, or (2) toward microglial subtypes ranging from *CD83^+^C1Q^hi^* microglia to *HSP^+^* microglia (Figure S3C). Overall, these data support the existence of circulating, mutually exclusive trajectories exhibiting macrophage or microglia properties and support the hypothesis that peripheral myeloid cells can adopt a microglia-like phenotype.^38^

Next, we validated the existence of these novel P2RY12-­expressing (microglia-like) peripheral myeloid populations using spectral flow cytometry in PBMCs isolated from IDH-WT, IDH-Mut glioma patients, or healthy subjects. Remarkably, we observed populations of cells resembling immunophenotypic microglia (CD45^mid^ CD11b^+^ P2RY12^+^) in both IDH-Mut and IDH-WT patient samples comprising around 1%-2% of viable cells. Circulating microglia-like cells were absent from healthy donor samples (Figure S3D-F). Interestingly, flow cytometry analysis captured a similar stratification of these cells with combinatorial expression of *C1Q* and *CD83* (Figure S3G). We then hypothesized that this phenotype may be induced by secreted factors derived from glioma cells. To that end, we cultured PBMCs isolated from healthy donors using either IDH-Mut or IDH-WT patient-­derived neurosphere conditioned media. Following 24 and 48 hours of co-culture, a consistent population of CD45^mid^ CD11b^+^ P2RY12^+^ myeloid cells emerged in all conditioned media-treated cells and were absent in the controls (Figure S4A-D). Finally, we successfully isolated this population from an IDH-Mut patient and found that it alters the expression of activation and degranulation markers (CD69 and LAMP-1) in NK-92 cells during co-culture with and without the presence of glioma neurospheres (Figure S5). Taken together, these data affirm the existence of P2RY12^+^ glioma circulating microglia-like cells (gCMGs) that are transcriptionally-aligned and immunophenotypically similar to intracerebral microglia and suggests that they are induced by tumor-secreted factors.

### Granzyme-Expressing T Cells Are Shared in All Genotypes of Glioma

**Figure 3.**
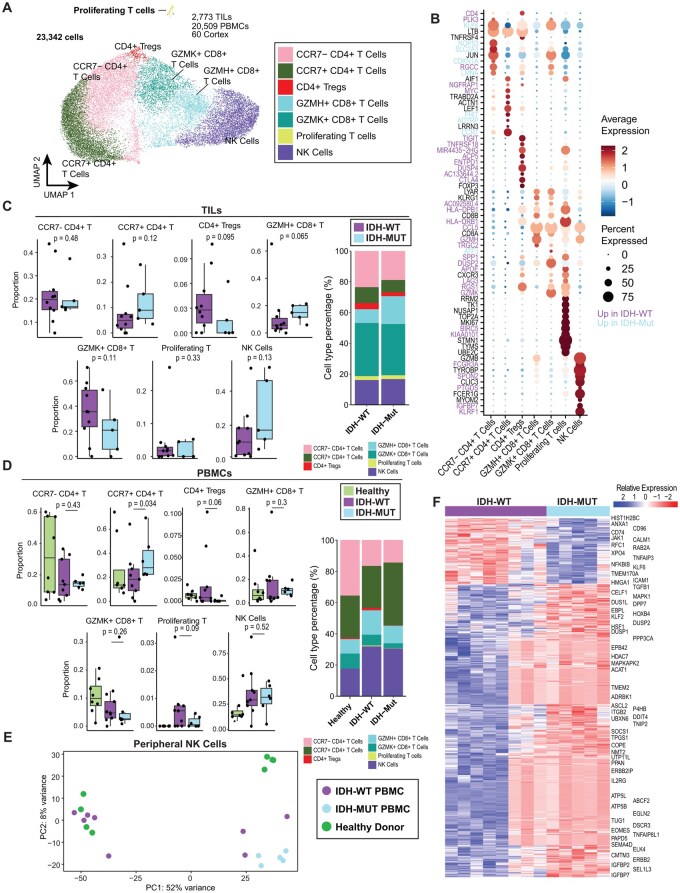
*CCR7^+^*CD4^*+*^ T cells predominate the peripheral lymphoid response to isocitrate dehydrogenase (IDH)-Mut glioma. (A) UMAP projection of lymphoid subclusters, showing 7 cell populations of T and NK cells (23 342 cells). (B) Dot plot showing expression of top 10 differentially expressed genes for each lymphoid cell type. *CD4* expression was included for reference. Dot sizes indicate the percentage of cells in each cluster expressing the genes, and colors indicate the relative average normalized expression level. Gene names are colored if they are differentially expressed (*P*-adjusted < 0.05) across all cells derived from IDH-WT versus IDH-Mut patients. (C)-(D) Composition of T/NK cell subtypes in (C) TILs and (D) PBMCs comparing IDH-WT and IDH-Mut gliomas. Left, boxplots showing the percentage of subtype type across samples as a fraction of all T/NK cells. Samples with at least 25 cells were included (TILs, *n* = 11 IDH-WT, 5 IDH-Mut; PBMCs, *n* = 9 IDH-WT, 6 IDH-Mut). Significance assessed using a one-sided Wilcoxon rank-sum test. Right, barplots showing differences in overall cell type distribution between IDH-WT and IDH-Mut glioma patients. (E) Principal component analysis (PCA) plot of pseudobulk RNA-seq profile of NK cells from PBMCs using all genes, demonstrating stratification into two main groups along the first principal component. Samples with at least 25 NK cells in the PBMCs were included (*n* = 8 IDH-WT, 5 IDH-Mut, 8 healthy donors). (F) Heatmap of differentially expressed genes between peripheral NK cells of IDH-WT and IDH-Mut glioma patients. Top differentially expressed genes are shown (*P*-adjusted < 0.05; 809 genes). Colors indicate pseudobulk expression values z-score normalized by row. Genes are ordered by hierarchical clustering. Samples are ordered by IDH mutation status.

Although gliomas are characterized by a paucity of lymphocyte infiltration, the identity of circulating T cells capable of cerebral transmigration and their genotype-dependencies remain unknown. Thus, we focused on annotating peripheral lymphoid subpopulations. After sub-clustering the T/NK cell populations, we identified 7 distinct lymphoid subtypes (Figure 3A, Figure S6A). Conventional CD4^+^ T cells (T_conv_) were primarily divided into two clusters stratified by expression of the lymphoid-homing *CCR7* chemokine receptor expressed in naïve (*T*_n_) and/or central memory (*T*_CM_) but not effector (*T*_eff_) T cells^39^,^40^ (Figure 3B, Table S4). Importantly, the *CCR7^+^* CD4^+^ T_conv_ cells exhibited high expression of *IL7R* as well as TCF7 and LEF1 regulons, supporting central memory T cell identity (Figure S2C-D, Table S5). Comparatively, *CCR7^-^* CD4^+^ T cells differentially expressed *LMNA, CDKN1A*, and AP1 factors (ie *JUN, FOS*) (Figure 3B), along with upregulation of KLF2 and NFKB1 regulon activity (Figure S2C-D). A substantial population of circulating CD8^*+*^ T cells was characterized by the diffuse granzyme gene expression (*GZMH* and *GZMK*). *GZMK*^+^CD8^+^ T cells expressed high levels of the chemokine *CCL5*, genes related to T cell activation (ie *HLA-DRB1/DPB1*) and the effector-related gene *KLRG1*, along with enrichment of an ASCL2 regulon shared with NK cells (Figure 3B, Figure S2C-D). *GZMK^+^*CD8^+^ T cells differentially expressed *RGS1*, *DUSP2*, and *DUSP4*, which have been identified as markers for T cell exhaustion in the TIME. A closely related T cell cluster (*GMZH*^+^CD8^*+*^ T cells) displayed fewer markers of exhaustion and greater levels of *GZMB*. The circulating immune compartment in gliomas also featured *FOXP3^+^* regulatory T cells (*T*_regs_) with high expression of immune checkpoint genes *CTLA4*, *TIGIT*, and *ICOS*, as well as a distinct population of proliferating T cells (Figure 3B).

Examining genotype-specific enrichment of T cells in the TIL scRNA-seq data revealed a trend towards higher abundance of both *CCR7^+^*CD4^+^ (naïve) T cells and *GZMH^+^*CD8^+^ T cells in the T/NK cell fraction in IDH-Mut glioma (Figure 3C). This observation is consistent with prior reports of distinct lymphocyte profiles between IDH-WT and IDH-Mut gliomas despite the overall immune-cold TIME of IDH-Mut glioma.^41^ Indeed, naïve *CCR7^+^*CD4^+^ T cells were significantly enriched in the T/NK cell subset of PBMCs in IDH-Mut patients (**Figure 3D**). Across all malignant gliomas observed in our cohort, *T*_regs_ comprised a small fraction of both infiltrating and peripheral T cells; however, the majority of *T*_regs_ in PBMCs were observed in IDH-WT patients (**Figure 3D**). Overall, these results highlight core shared and unique characteristics of peripheral T cell responses to IDH-WT and IDH-Mut glioma.

### TNF-α Signaling is Upregulated in IDH-Mut Glioma-Associated Peripheral Lymphoid Cells

NK cells are the central antigen-agnostic effector lymphocyte in the human immune system.^42^ We therefore set out to understand whether functional gene expression differences are observed in our cohort. Our scRNA-seq analyses detected a relatively homogeneous NK population primarily seen in PBMCs (Figure 3A-B). To overcome the sparsity of the scRNA-seq and characterize the differences in glioma-associated peripheral NK cells across genotypes, we applied pseudobulk analysis, pooling peripheral NK cells from each patient or healthy donor with at least 25 NK cells. Principal component analysis (PCA) revealed that the NK cells stratified into two main groups along PC1, encompassing the majority of the variance (Figure 3E). An unexpected finding was that peripheral NK cells from IDH-Mut glioma clustered tightly, whereas NK cells from IDH-WT glioma were dispersed with healthy donor NKs. Next, we calculated differentially expressed genes between the IDH-WT and IDH-Mut associated NK cells and identified 809 differentially expressed genes (FDR < 0.05, Figure 3F). Peripheral NK cells from IDH-Mut gliomas were enriched in genes related to metabolism (ie, *ATP5J, ATP5G2*), calcium signaling (ie, *ANXA1*, *CALM2*), and TNF-α signaling (ie, *TNFAIP8L1, TNFAIP3, NFKBIB*) (Figure 3F). Gene set enrichment analysis revealed differences in multiple metabolic pathways as well as significant upregulation in the *TNF-α signaling via NF-κB* pathway in IDH-Mut versus IDH-WT PBMCs (Figure 6A-C). These results suggest that glioma-associated peripheral NK cells do not form distinct clusters but exhibit IDH status-specific metabolic processing and inflammatory pathways.

Extending this analysis to other populations, we found that peripheral T cells in IDH-Mut patients also differentially expressed TNF-α signaling and inflammatory response pathways, particularly within CD8^+^ T cells (S7A-F). Additionally, we found that peripheral monocytes are split along PC2 between healthy donors and all glioma patient PBMCs, suggesting that the glioma state globally alters the transcriptional properties of peripheral myeloid cells (Figure S8A). Moreover, peripheral monocytes in IDH-Mut patients also differentially expressed inflammatory response pathways, including interferon signaling and antigen presentation as the top differentially expressed pathways. Lastly, we observed similar findings in intratumoral myeloid cells (ie, macrophages and microglia) (Figure S8B-F). Taken together, these results indicate that IDH-Mut glioma differentially alters both myeloid and lymphoid populations in favor of an inflammatory state.

### Validation of Transcriptional Populations with Multispectral Flow Cytometry

To validate the distribution of populations identified via scRNA-seq and confirm the presence of protein expression across important cell subsets, we performed flow cytometry on TILs and PBMCs isolated from IDH-WT and IDH-Mut donors using a panel of key markers driven entirely by our sequencing data (Table S6). This analysis confirmed discrete lymphoid subsets driven by the expression of *CCR7*, *GZMK*, and *GZMH*, as well as myeloid subsets driven by both *CD83* and *C1Q* expression (Figure S9A-L). Notably, we found that *CD83*/*C1Q-*coexpressing subsets were enriched in IDH-Mut samples both intratumorally and in the peripheral blood (Figure S9E-F, K-L). This protein-based data are consistent with our single-cell genomic analyses and therefore supports our findings in which *CD83*, *C1Q*, *CCR7* and granzyme genes define specific genotype-predominant subsets.

### Emergence of an Immune Signature That Predicts Overall Survival in a Genotype-Independent Manner

**Figure 4.**
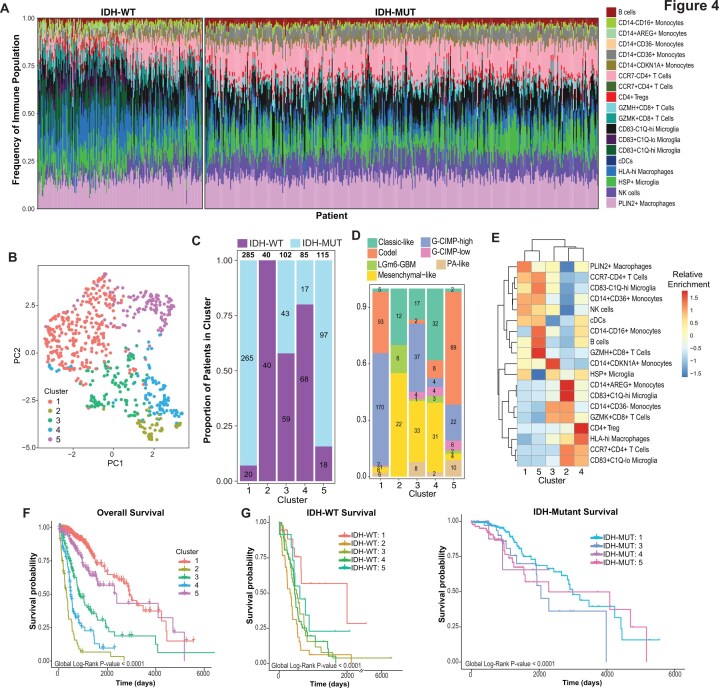
Deconvolution of bulk glioma transcriptomes reveals distinct clusters of patients based on immune cell frequencies. (A) Quantification of relative immune cell frequencies from TCGA using CIBERSORTx deconvolution. Samples are split by IDH mutation status. (B) Principal component analysis (PCA) based on inferred immune cell frequencies revealed 5 clusters of patients, derived via k-means clustering. (C) Relationship between immune clusters and IDH mutation status. The number of patients in each category is indicated. (D) Relationship between immune clusters and DNA methylation-based classifications.^5^ Number of patients in each category is indicated. (E) Enrichment of immune cell signatures in each cluster. Colors indicate deconvoluted cell type proportion z-score normalized by row. (F) Survival curves demonstrate significant differences in overall survival between the 5 immune signature clusters. Significance was assessed with a global log-rank test. (G) Survival curves of patients within each immune signature cluster stratified by IDH mutation status. Significance was assessed with a global log-rank test.

Overall, our data confirm shared immune cellular profiles but varying genotype-dependent cellular proportions/frequencies across IDH-WT and IDH-Mut glioma. These data support the previously observed finding that cumulative composition of immune cells converge to induce differential immune subtypes across glioma states. However, it is unknown whether cumulative immune effects contributed by variable immune cell composition affect survival in an entirely genotype-dependent way or whether patient survival is independently attributable to antitumor immunity. To capture distinct immunologic clusters in glioma, we applied CIBERSORTx using our scRNA-seq TIL data as a reference dataset to deconvolute the relative frequencies of immune subtypes identified across bulk-RNAseq transcriptomic profiles from the TCGA database. We applied this method across the TCGA glioblastoma cohort (TCGA-GBM) and diffuse-grade glioma cohort (TCGA-LGG), including 627 patients (Figure 4A). We observed significant heterogeneity across the patients and identified recurring patterns of immune signatures. Unsupervised clustering based entirely on immune signatures inferred 5 discrete immunologic clusters (Figure 4B). Clusters 2 and 4 were composed primarily of IDH-WT samples, while clusters 1 and 5 were composed primarily of IDH-Mut samples. Notably, cluster 3 was comprised of similar proportions of IDH-WT and IDH-Mut samples (Figure 4C). We also observed differences in the distribution of DNA methylation-based subtypes^5^ across the immunologic clusters (Figure 4D).

Deeper examination of these novel immunologic clusters revealed that IDH-WT-predominant clusters (2 and 4) were most enriched in T_reg_ immune cellular signatures (Figure 4E, Figure S10), whereas the IDH-Mut-predominant clusters (1 and 5) were enriched in monocyte, NK cell, and *HSP*^+^ microglia signatures. Interestingly, the mixed cluster (3) was enriched for the *GZMK^+^*CD8^+^ T cell signature described above, supporting the existence of shared granzyme-expressing lymphocyte ontogenies initially seen in our cohort-specific analysis (Figure 4E, Figure S10). Notably, *GZMK^+^*CD8^+^ T cells were one of few immune cell types to exhibit significant correlation between PBMCs and TILs, suggesting that they can populate both circulating immune and tumor-infiltrating (TIL) compartments in malignant glioma (Figure S11). Next, we assessed overall survival (OS) across these 5 immunologic clusters. Unsurprisingly, we found that clusters 2 and 4 had the lowest OS, and clusters 1 and 5 had the highest OS, given the IDH-WT and IDH-Mut predominance, respectively (Figure 4F, Figure S12). Notably, clusters 2 and 4 exhibited both the highest enrichment of *T*_reg_ signatures and the lowest OS, but are also represented by the highest fraction of IDH-WT samples. This could indicate the presence of an effect beyond IDH, in which *T*_reg_-driven immunosuppression may harbor unique immunologic morbidity in glioma.

To confirm that immunologic signatures remain pertinent predictors of OS in the absence of genotype, we stratified OS within the IDH-WT and IDH-Mut cohorts in the TCGA dataset. Remarkably, the prognostic effects of immunologic clusters remained relevant despite controlling for the prognostic effects of IDH-Mut genotypes from the analysis. Indeed, within the IDH-WT TCGA cohorts, patients in cluster 2 had the shortest OS, patients in cluster 1 had the longest OS, and patients in clusters 3 and 4 had an intermediate OS. For the IDH-Mut patients, cluster 3 had the shortest survival, whereas patients in cluster 1 had the longest OS (Figure 4G, Figure S13). Taken together, our data suggest that immune transcriptomic signatures exert prognostic effects on overall survival independent of IDH status. An important consequence of this finding is that immune phenotypes gleaned from peripheral blood may be predictive of intratumoral immunologic states and, by extension, overall prognoses.

**Figure 5.**
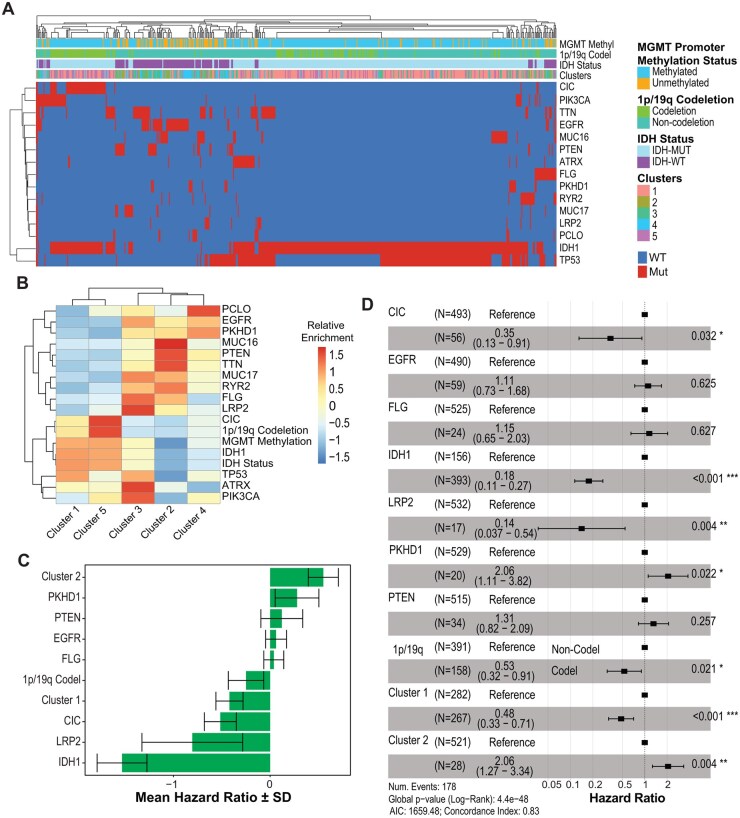
Immune-based transcriptomic signatures are independent prognostic indicators in high-and low-grade glioma patients. (A) Heatmap showing the relationships between common somatic mutations and immune clusters described in Figure 5. Samples are labeled by their immune cluster number and colored by whether they have a mutation in the indicated gene. MGMT methylation status, 1p/19q codeletion status and isocitrat dehydrogenase (IDH) mutation status are indicated. Samples are ordered using hierarchical clustering. (B) Heatmap showing the overall proportion of frequent somatic mutations in each of the 5 clusters. Data is *z*-score normalized by row. (A-D) Elastic net cox-proportional hazard analysis based on 10-fold cross-validation jointly utilizing somatic mutations and immune signature clusters identifies the relative contribution of each individual variable to overall survival. Higher values indicate a worse survival outcome, and lower values indicate a more favorable survival outcome. Results are shown for variables inferred to be most significant across cross-validation folds.

Given the known prognostic impact of glioma-wide somatic genetic alterations, including *EGFR* and *ATRX*, we examined the prevalent genetic alterations within each immune-based cluster. We identified that clusters 1 and 3 had the highest frequency of *TP53* mutations, cluster 2 had the highest frequency of *PTEN* mutations, cluster 4 had the highest frequency of *EGFR* mutations, and cluster 3 was enriched for mutations in *PIK3CA, ATRX*, and *LRP2* (Figure 5A-B). To further examine the effects of granular genomic features beyond IDH status, we applied a Cox proportional hazards elastic net regression model to identify the relative contribution of individual somatic mutations and immune cluster variables. This analysis confirmed the prognostic importance of *IDH1* mutations, and highlighted previously established prognostic mutations, such as *PKHD1.*^43^ Importantly, this analysis implicates cluster 1 (low risk) and cluster 2 (high risk) as significant independent immunologic prognostic indicators even when controlling for common glioma-specific somatic mutations including IDH1/2, p53, EGFR, PTEN, ATRX, MGMT methylation status, and 1p/19q co-deletion (Figure 5C-D). These analyses demonstrate that intratumoral immune signatures derived via the single-cell transcriptomic data yield independent prognostic indicators that predict overall survival across glioma subtypes.

### Cell-Cell Interaction Analysis Predicts Intercellular Communication Between Intratumoral and Peripheral Immune Populations

**Figure 6.**
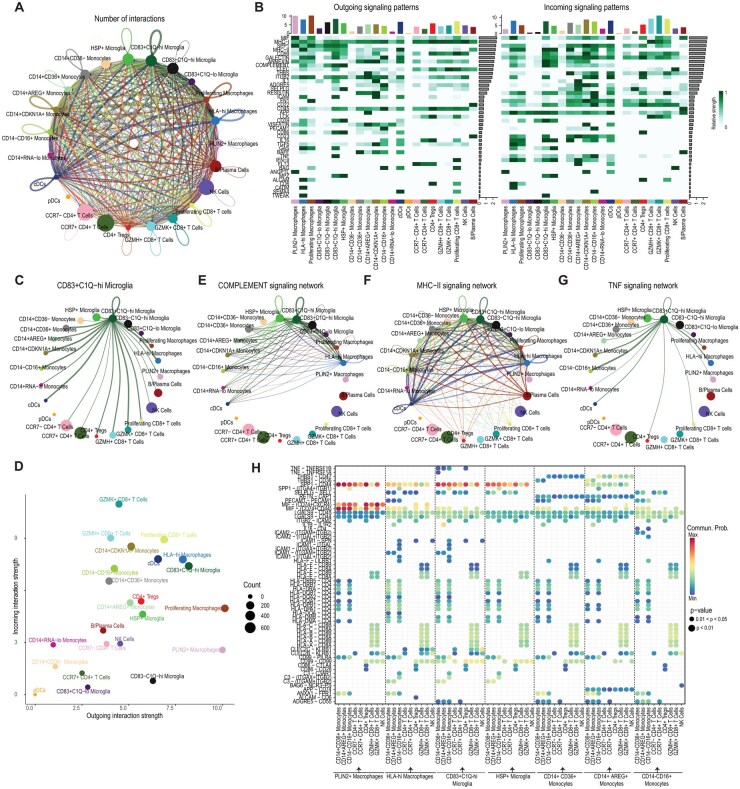
Inference of intercellular communication between immune subtypes. (A) Aggregated cell-cell communication network as inferred by CellChat. Dot size indicates the relative number of cells of each cell type, and line thickness connecting cell types indicates the number of interactions between each cell type pair. All data were included in the analysis (peripheral blood mononuclear cells, tumor-infiltrating leukocytes, non-neoplastic cortex) from both genotypes and healthy donors. (B) Significant outgoing (ligand enriched) and incoming (receptor-enriched) signaling predictions were scored for each annotated signaling pathway, and the strength of signaling (edge weights) for each cell type was scaled and plotted by pathway. The top barplot shows the total signaling strength of a cell group by summarizing all signaling pathways. The right barplot shows the total signaling strength (network edge weights for that group of ligand-receptor pairs) across all cell groups, illustrating whether a pathway is broadly activated. (C) Signaling sent from *CD83^+^C1Q^hi^* microglia. Dot size indicates relative cell abundance as in (A), and line thickness connecting cell types represents the total interaction strength (edge weights) between that cell type pair. (D) Relative contributions of each cell type to the cell-cell communication network. Dot size is proportional to the number of inferred links (either outgoing or incoming) associated with each cell group. (E)-(G) Network diagram of inferred signaling for (E) Complement, (F) MHC-II, and (G) TNF signaling pathways. Dot size indicates relative cell abundance as in (A), and line thickness represents the strength of the interaction, aggregating edge weights for that group of ligand-receptor pairs. (H) Significant ligand-receptor pairs between selected cell types. Dot color represents the calculated communication probability, and dot size represents *P* value of the interaction.

Finally, to better understand the roles of the immune subpopulations from our integrated analysis, we utilized CellChat to infer patterns of intercellular ligand-receptor interactions across all myeloid and lymphoid subsets identified in this study. We utilized our full integrated scRNA-seq data across the 23 cell subtypes for this analysis. Given the multifactorial roles of immune cells in the tumor microenvironment and peripheral immune system, we observed a complex network of cell-cell interactions involving every cell type (except pDCs due to low abundance) (Figure 6A). We examined the signaling pathways that were involved in these interactions and found pathways known to be significant in glioma, such as the MIF and SPP1 pathways,^44^ along with various chemokine interactions and antigen presentation pathways in which most cell types were involved (Figure 6B, Figure S14A-F). Given the high expression of complement genes in several microglia populations, it is unsurprising that we also uncovered the complement pathways as a top contributor to intercellular crosstalk.

Given these findings, we further characterized the roles of *CD83^+^C1Q^hi^* microglial cells enriched in IDH-Mut glioma and unexpectedly present in the PBMCs of glioma patients. Intriguingly, we found that *CD83^+^C1Q^hi^* microglia were inferred to interact widely with both myeloid and lymphoid populations, with most interactions predicted to occur with *GZMK^+^*CD8^+^ T cells as well as monocyte subpopulations, including *CD14*^–^*CD16^+^* monocytes and *CD14^+^CD36^+^* monocytes (Figure 6C). *CD83^+^C1Q^hi^* microglia also had a high outgoing interaction strength relative to other populations (Figure 6D). The other populations with relatively high levels of outgoing interactions included *PLIN2^+^* macrophages, proliferating macrophages, and *HLA^hi^* macrophages. The populations with the highest levels of incoming interactions included all CD8^+^ T cell populations as well as several monocyte populations. Interestingly, CD4^+^ T cells were relatively inert in both their outgoing and incoming interactions (Figure 6D). These findings suggest that myeloid populations exert unusually potent effects on adaptive antitumor immunity in the glioma tumor microenvironment.

*CD83^+^C1Q^hi^* microglia were found to signal through multiple pathways. For example, along with *HSP^+^* microglia, they were predicted to communicate through the complement pathway. Along with *HLA^hi^* macrophages and dendritic cells, *CD83^+^C1Q^hi^* microglia exhibit the genomic machinery necessary for antigen presentation, including MHCII and CD80 interactions (Figure 6E-F, Figure S14A-B). Uniquely, *CD83^+^C1Q^hi^* were inferred to be the only significant sender of the pro-inflammatory TNF pathway, through which nonclassical *CD14^-^CD16^+^* monocytes were the primary receiver through expression of *TNFRSF1* (TNFR2) (Figure 6G-H, Figure S14C). Nonclassical monocytes were rare in the TIME and were found predominantly in the PBMCs of IDH-Mut glioma patients (Figure 2D-E). Finally, we found similar observations when the analysis was run on subsets stratified by IDH mutation status and compartment (Figures S15 and 16). This highlighted the central role of *CD83^+^C1Q^hi^* microglia in the TIL compartment versus the predominant role of *CCR7^+^*CD4^+^ T cells in the PBMCs in both genotypes. Taken together, this analysis infers differential patterns of intercellular communication within IDH-WT and IDH-Mut glioma and distinguishes cellular components shared or enriched within genotypes that underlie immune-dependent signaling.

## Discussion

The current study profiles granular intratumoral and peripheral immune cells at the single-cell level across IDH-WT and IDH-Mut glioma using CD45-sorted cells to investigate immunologic transcriptional signatures. The depth, granularity, and paired nature of our analyses uncovered both restricted and shared immune signatures within distinct immune compartments and genotypes. Within the circulating immune cells, our results suggest that IDH-Mut gliomas possess a lymphoid-predominant immune response enriched in CD4^+^ T cells, while IDH-WT gliomas possess a more heterogeneous monocyte/macrophage enrichment.

Unexpectedly, we identified the presence of microglia-like myeloid cells in the PBMCs of glioma patients, but not healthy donors, and predicted their ability to interact broadly with other immune subtypes. Given the ability of myeloid cells to cross the blood-brain barrier, it is possible that these microglia-like cells are tissue-resident microglia migrating across the glioma blood-brain barrier and into the peripheral blood. However, it has been previously demonstrated that peripheral blood myeloid cells can adopt microglia-like phenotypes,^11^^,^^38^^,^^45^ and the induction of P2RY12-expressing cells upon treatment with neurosphere-conditioned media supports the hypothesis that stromal tumor compartments are not required for circulating microglia-like cell formation. While we cannot exclude the possibility that technical artifacts from barcode swapping between samples^46^ contributed to this observation, the orthogonal observation with flow cytometry ameliorates this concern. Future studies are required to clarify the origin, functional significance and ontogenesis of these cells; however, their presence may have diagnostic and prognostic significance. Importantly, while they are transcriptionally and immunophenotypically similar to canonical microglia, future work will determine if they can assume the full spectrum of functional activities of typical microglia.

The expression of *CD83* on glioma-associated microglia has been previously reported.^47^ In other cellular contexts, microglial *CD83* expression has been associated with pre-activated microglia or early activation during neuro-inflammation,^48^^,^^49^ yet its functional role in glioma has not been directly elucidated. A recent study demonstrated that *CD83* directly modulates the reaction to neuro-inflammatory damage in the central nervous system, with *CD83*-deficient microglia exhibiting an excessive pro-inflammatory response,^50^ suggesting that *CD83* mediates both cellular activation and the resolution of inflammation. Taken together, these results indicate the possibility that *CD83* expression in glioma-associated microglia may be responsible for the differential immune environment observed in IDH-Mut glioma. Our data nominate *CD83* as a potential subtype-specific immunotherapeutic target in glioma.

In addition to the identification of immune profiles within TIL and PBMC compartments of glioma, we found that composite immunologic signatures may be independent prognostic indicators across IDH-Mut and IDH-WT glioma. By deconvoluting bulk RNA-seq data using only immune cell subtypes, we identified clusters of patients with differential overall survival independent of IDH mutation status as well as other common somatic mutations. We utilized immune cell profiles to demonstrate the clinical significance of immune-based transcriptomic signatures. Since immune cells comprise a minority of the glioma microenvironment, this data does not purport to characterize immune-stromal and immune-tumor interactions that facilitate important molecular signals that occur throughout gliomagenesis and maintenance.^30^^,^^47^ Thus, additional profiling at the single-cell level comparing our immune-centric analysis to stroma/tumor-directed annotations will ultimately be required to determine distinct effects of tumor-immune composition on patient outcomes. Nevertheless, the immunologic focus of this study enables the elucidation of unique TIME features that may lead to flexible, personalized immunotherapeutic strategies.

While our study highlights intratumoral and peripheral immune states in IDH-WT and IDH-Mut glioma, futures studies are needed to define the roles of these distinct subpopulations, especially with regard to molecular mechanisms underlying the pathogenesis in glioma. This would include functional studies on isolated peripheral and intratumoral populations as well as spatial profiling of the glioma microenvironment, which could effectively capture tumor-immune signaling interactions not assessed by our ligand-receptor analysis restricted to immune populations. We are particularly interested in examining the immunosuppressive potential of *CD83*^+^ microglia in IDH-Mut glioma, the developmental mechanisms and roles of microglia-like cells in the peripheral blood of glioma patients, and their effects on T cell transcriptional profiles and function. Finally, our study nominates a variety of peripheral immune states which can be validated in future studies to provide sensitive, specific, and clinically predictive biomarker diagnostics across glioma genotypes.

## Supplementary Material

noaf206_Supplementary_Data

## Data Availability

Raw demultiplexed sequencing data and processed feature/barcode matrices are available on the Gene Expression Omnibus (NCBI GEO) under accession number GSE247824. No new software was created for this study. Standard pipelines and analytical packages were employed as previously described. Any additional information required to reanalyze the data reported in this paper is available from the lead contact upon request: Nduka Amankulor (nduka.amankulor@pennmedicine.upenn.edu).
