# Corrigendum to: Single-cell profiling of peripheral and local immune compartments reveal unique genotype-independent prognostic immune signatures across isocitrate dehydrogenase-stratified glioma

**DOI:** 10.1093/neuonc/noag092

**Published:** 2026-05-09

This is a corrigemdum to: Jonathan H Sussman, Anthony R Cillo, Sajeev Kohli, Carly Cardello, Jonathan Patterson, Ebrar Akca, Angelo Angione, David Moon, Katharine Krueger, Ali Ghamari, Emily Xu, Jessica Xu, Antonio C Tarbay, Alex Li, Bhargavi R Budihal, Xiaoran Zhang, Omar Elghawy, Wojciech K Panek, Prajwal Rajappa, Kai Tan, Dario A A Vignali, Tullia C Bruno, Nduka M Amankulor, Single-cell profiling of peripheral and local immune compartments reveal unique genotype-independent prognostic immune signatures across isocitrate dehydrogenase-stratified glioma, *Neuro-Oncology*, Volume 28, Issue 1, January 2026, Pages 143–158, https://doi.org/10.1093/neuonc/noaf206

There was an error on Figure 4E, where the cluster labels below the heatmap were missing. This is now corrected in this figure within the article.

**Addendum 30 July 2026:** In the original publication, the **Supplementary data** files were incomplete and the supplementary tables were omitted. Files containing both the ­supplementary figures and tables have now been published with the article.

