# Supplementary material for: Single-cell profiling of peripheral and local immune compartments reveal unique genotype-independent prognostic immune signatures across isocitrate dehydrogenase-stratified glioma
Source: Neuro Oncol. 2025 Sep 23;28(1):143–58. doi: 10.1093/neuonc/noaf206 (PMC12962638; doi:10.1093/neuonc/noaf206)
Supplement: noaf206_Supplementary_Data [file noaf206_supplementary_data.zip › Supplementary_Figs_Method.docx]

**Single-cell profiling of peripheral and local immune compartments reveal unique genotype-independent prognostic immune signatures across isocitrate dehydrogenase-stratified glioma**

**Supplementary Figures 1-7** and **Supplemental Methods**

**
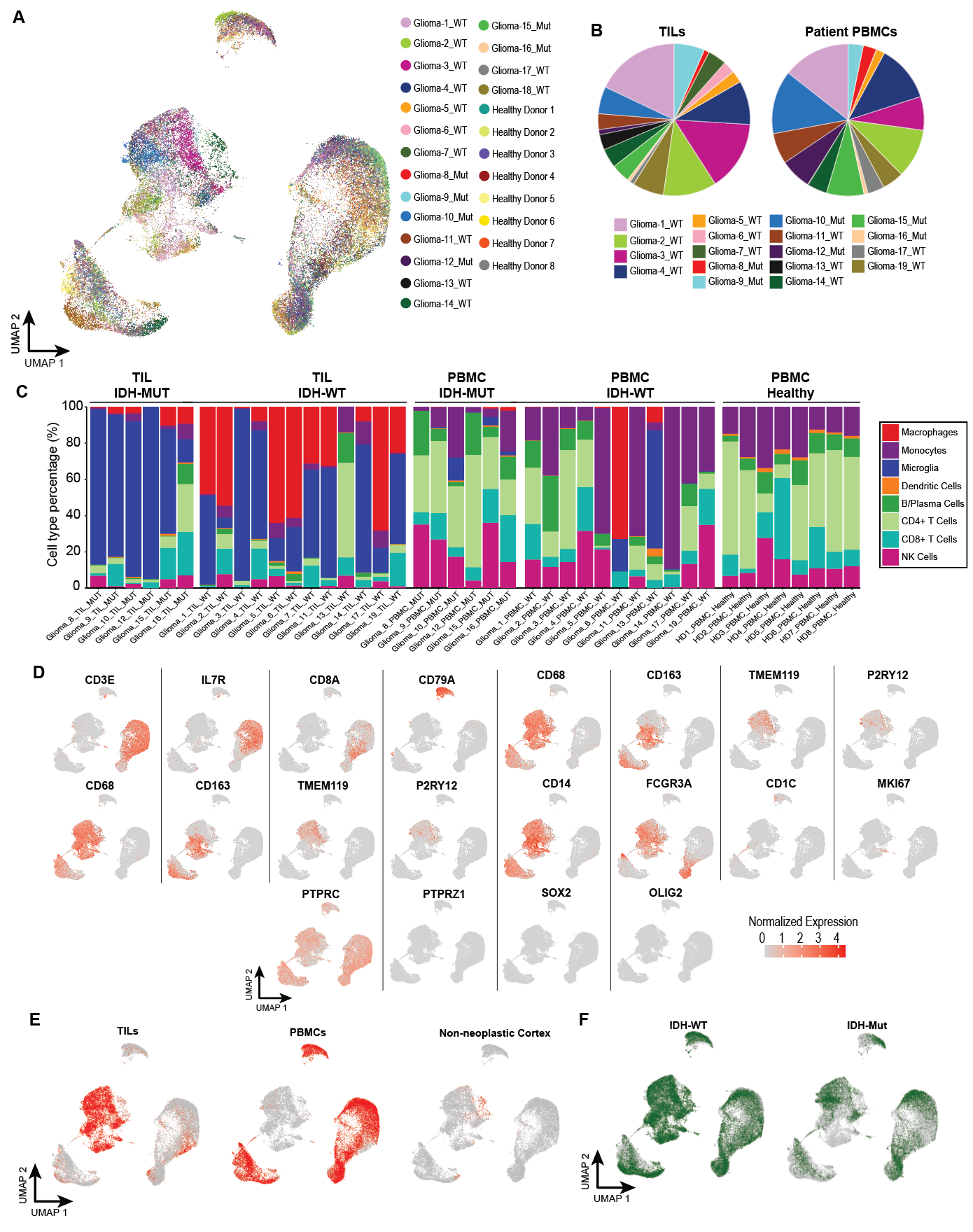
**

**Supplementary Figure 1.** **Integration and annotation of scRNA-seq data.** **(A)** UMAP of scRNA-seq dataset colored by sample, demonstrating minimal batch effects between samples. HD, healthy donor. **(B)** Pie chart demonstrating contribution of each patient to the intratumoral and peripheral immune profiling. TIL, tumor infiltrating leukocyte; PBMC, peripheral blood mononuclear cell. **(C)** Barplot showing distribution of course cell types in each sample included in the dataset. **(D)** UMAP colored by expression of selected marker genes used for annotation of coarse cell types. **(E)** UMAP plots with cells derived from the tumor (TILs), peripheral blood (PBMCs) and adjacent non-neoplastic cortex highlighted. **(F)** UMAP plots with cells derived from IDH-WT and IDH-Mut glioma patients highlighted.


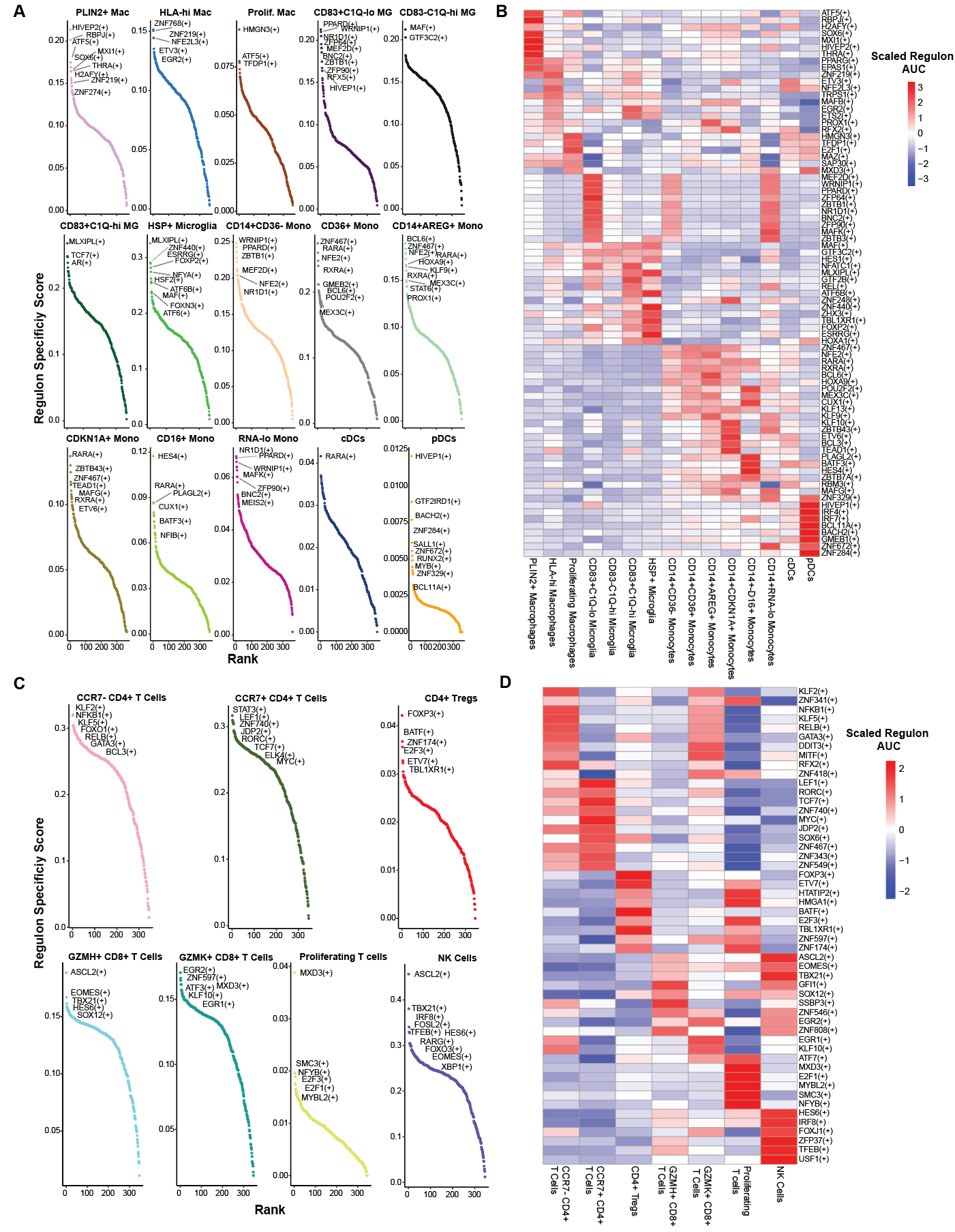


**Supplementary Figure 2. Transcriptional regulons of myeloid and lymphoid subtypes. (A)** Waterfall plots of regulon specificity scores for each subpopulation of myeloid cells. Top 5-10 selected regulons are indicated for each population. **(B)** Heatmap of regulon AUC values showing top 10 differentially active regulons for each myeloid subtype. Values are z-score normalized by row. **(C)** Waterfall plots of regulon specificity scores for each subpopulation of lymphoid cells as in (A). **(D)** Heatmap of regulon AUC values showing top 10 differentially active regulons for each lymphoid subtype as in (B).

**
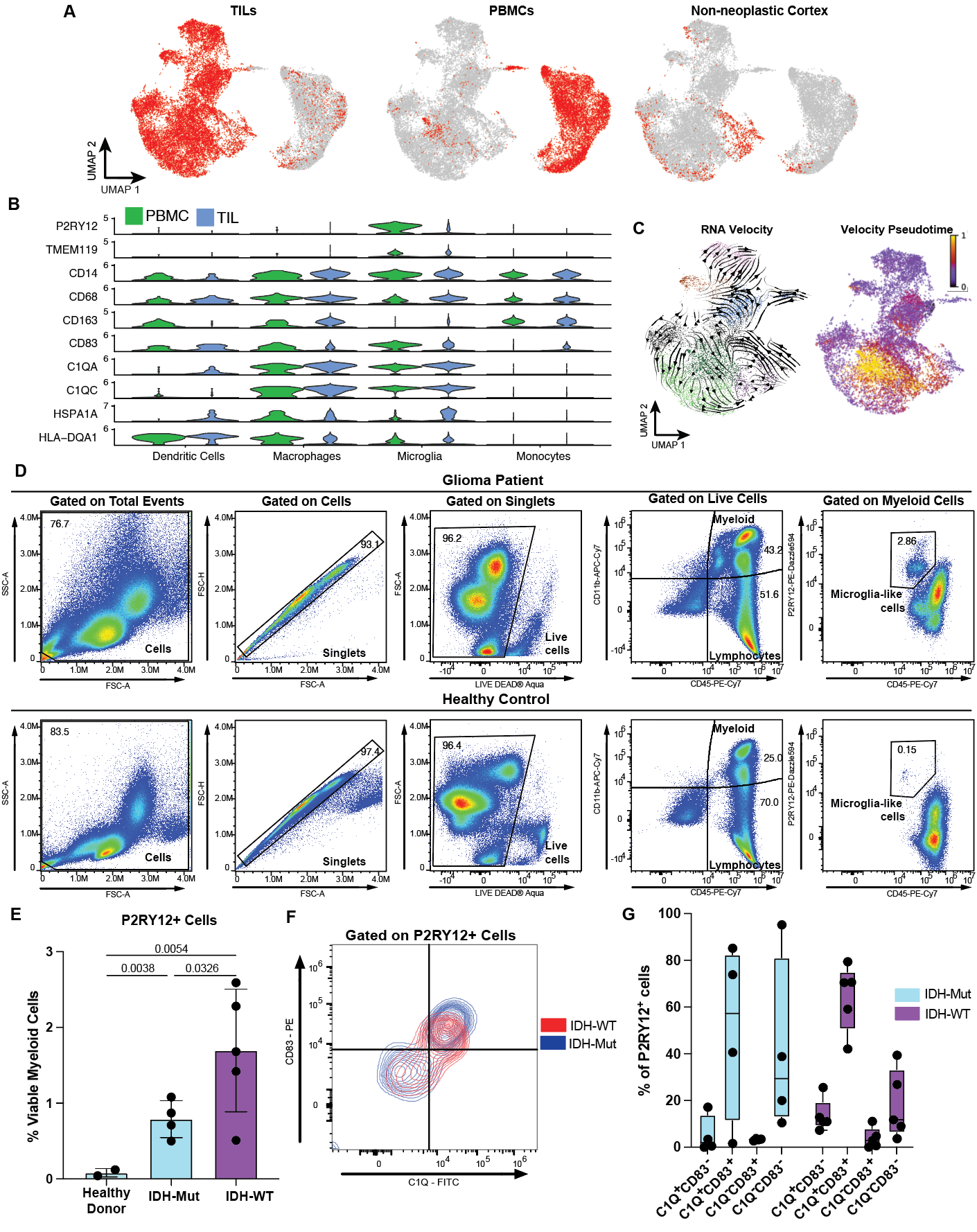
**

**Supplementary Figure 3. Identification of P2RY12^+^ cells in patient PBMCs. (A)** UMAP plots with cells derived from the tumor (TILs), peripheral blood (PBMCs), and adjacent non-neoplastic cortex highlighted. **(B)** Violin plots of normalized scRNA-seq gene expression of myeloid marker genes stratified by PBMCs from glioma patients and TILs. Cells are groups by their coarse annotation. **(C)** Left, RNA velocities and associated vector fields across all myeloid populations. Right, inference of pseudotime based on the vector field in the left panel. **(D)** Gating strategy for flow cytometry analysis of patient PBMCs, showing representative samples from a glioma patient and healthy control. A population of CD45^mid^ P2RY12^+^ cells is highlighted as a microglia-like population. **(E)** Quantification of P2RY12^+^ myeloid cells as a fraction of viable myeloid cells isolated per sample from 2 healthy donors, 4 IDH-WT patients, and 5 IDH-Mut patients. Significance was assessed via the one-sided Welch’s t-test. **(F)** Expression of CD83 and C1Q by P2RY12^+^ myeloid cells in PBMCs isolated from 5 IDH-WT patients, and 4 IDH-Mut patients. Cells were pooled based on IDH-subtype. **(F)** Proportions of P2RY12^+^ myeloid cells based on their expression of CD83 and C1Q in (**E**), stratified by IDH subtype.

**
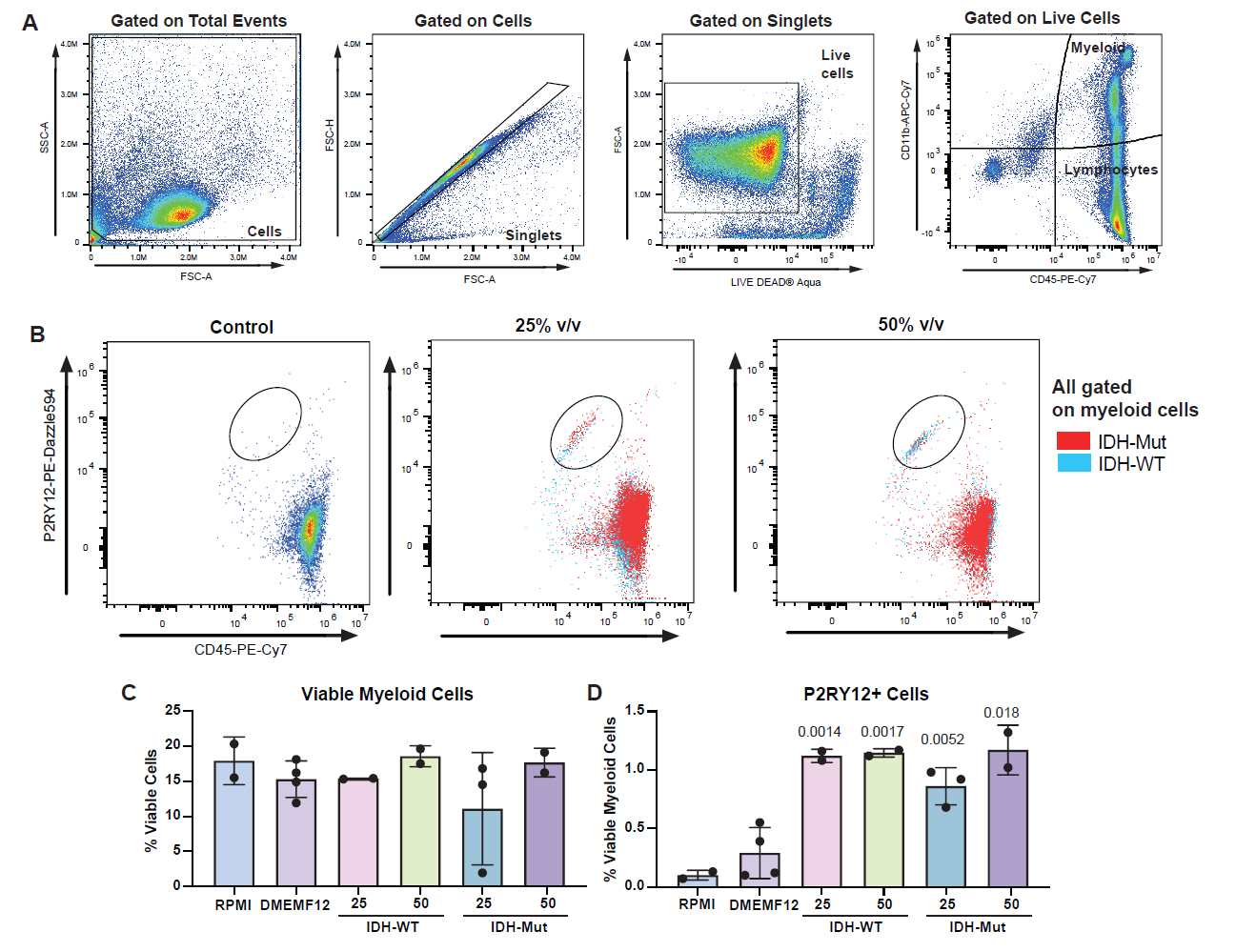
**

**Supplementary Figure 4. Glioma neurosphere conditioned media induces a P2RY12^+^ population in cultured PBMCs. (A)** Gating strategy for flow cytometry analysis of cultured PBMCs from one representative sample. **(B)** Examples of CD45^mid^ P2RY12^+^ cells identified in myeloid fraction of conditioned media-treated PBMCs (circled). Cultures were treated for 24 hours with 25% volume/volume (v/v) or 50% v/v neurosphere conditioned media from IDH-WT or IDH-Mut patient-derived neurosphere cultures or control media (either RPMI or DMEM/F12). Myeloid cells were identified based on CD11b expression. **(C)** Proportion of viable myeloid cells as a fraction of all viable cells in each sample. **(D)** Proportion of P2RY12^+^ cells in myeloid compartment of each sample as identified in (B).


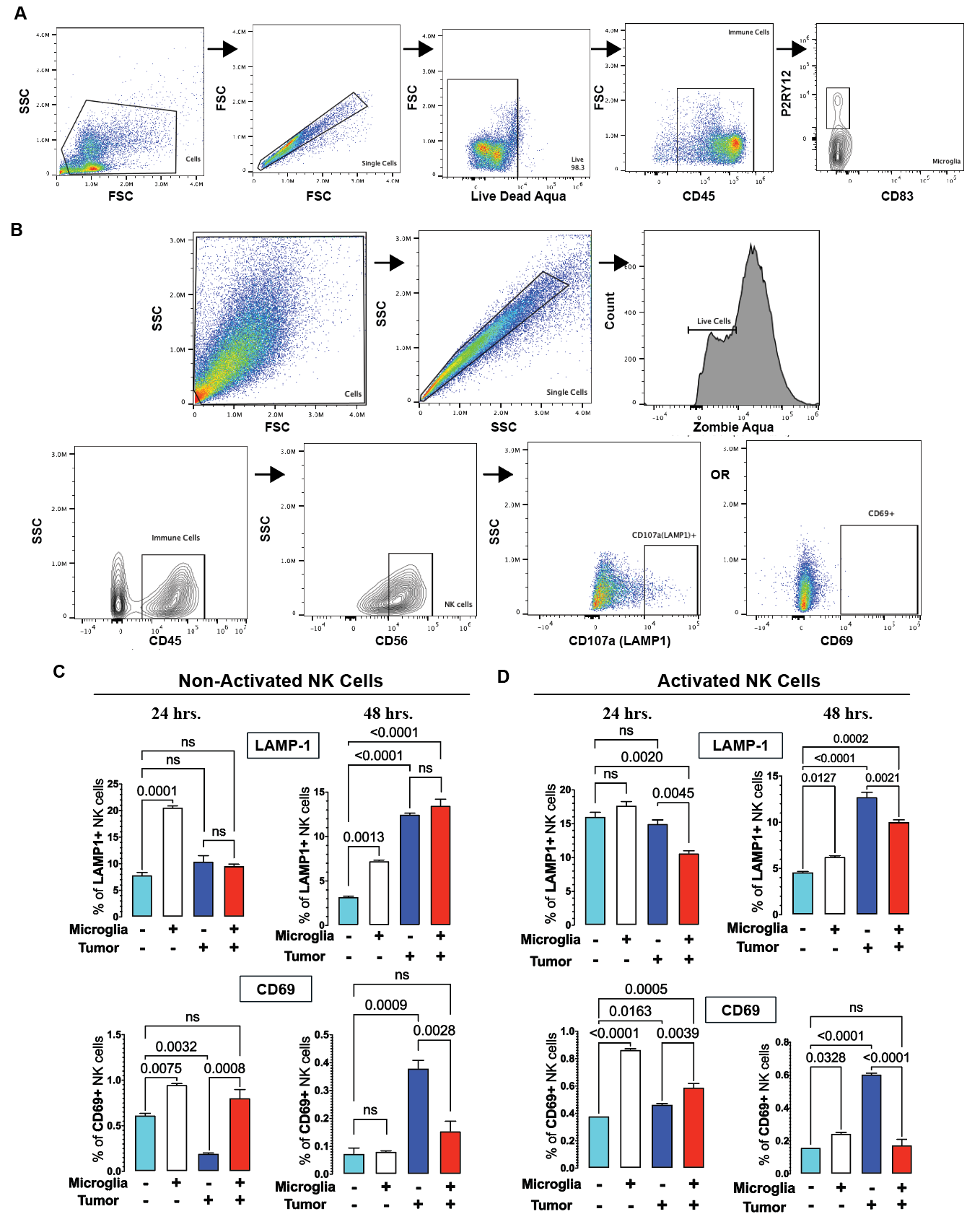


**Supplementary Figure 5. Peripheral microglia-like cells from IDH-mutant patient suppress NK-92 cell activation and degranulation in a cytokine- and time-dependent manner. (A)** Gating strategy to isolate peripheral microglia-like cells. **(B)** Gating strategy to quantify surface marker expression on NK-92 cells following co-culture. **(C-D)** LAMP-1 and CD69 expression were analyzed in NK-92 cells that were pre-cultured with microglia-like cells for 24 or 48 hours under both (C) cytokine-free and (D) cytokine-rich (+IL2/+IL15) conditions, then exposed to IDH-mutant glioma tumor cells for 2 hours. These preliminary findings reveal that subacute exposure of NK-92 cells to microglia (48h) significantly reduces both LAMP-1 and CD69 expression, while acute exposure (24h) induces mild, but statistically significant upregulation of both markers. Data represent mean ± standard deviation; ns, not significant (p > 0.05) by one-way ANOVA. N=2 technical replicates.

**
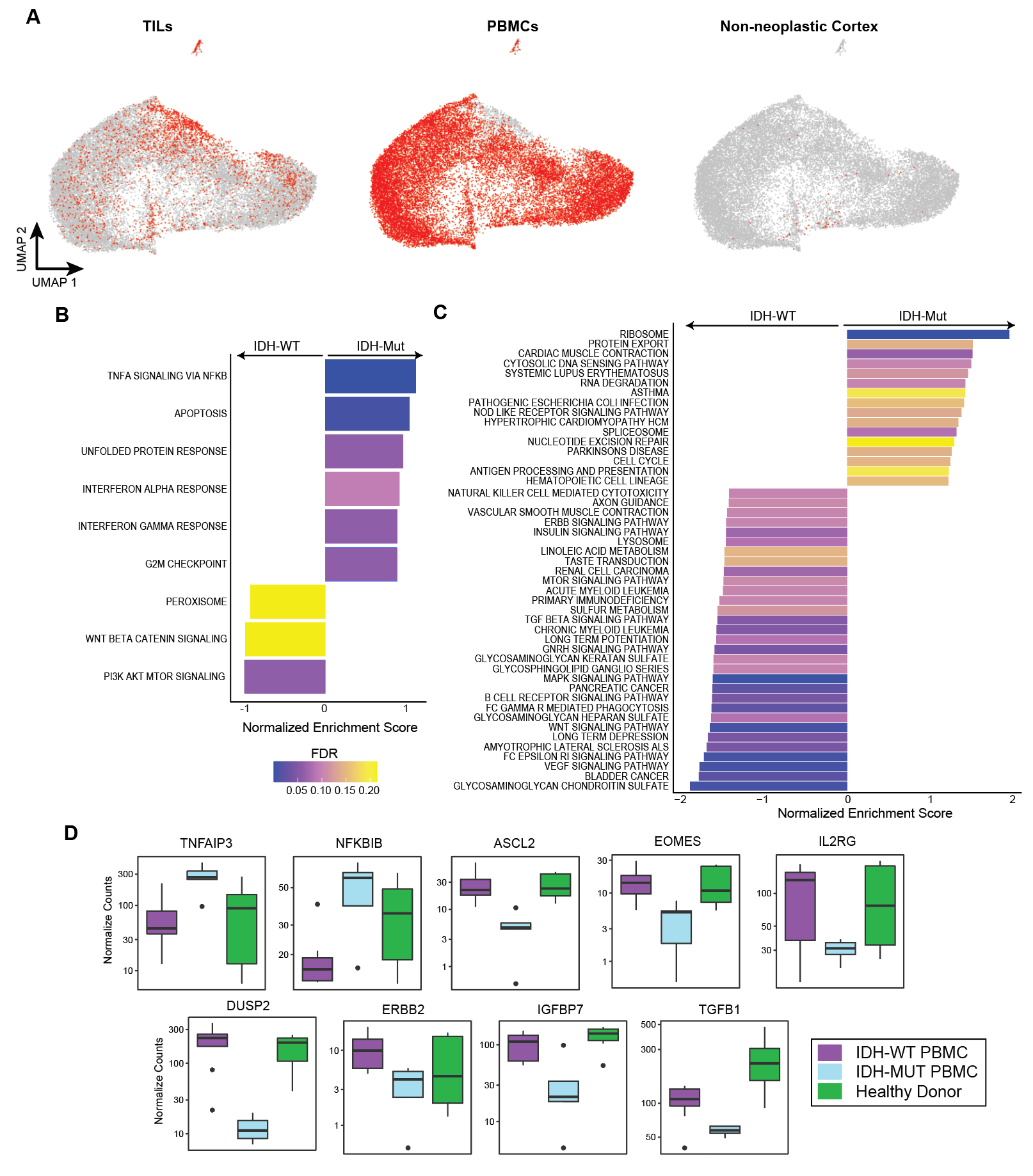
**

**Supplementary Figure 6. Pathway analysis of pseudobulk NK cell populations. (A)** UMAP plots with cells derived from the tumor (TILs), peripheral blood (PBMCs), and adjacent non-neoplastic cortex highlighted. **(B-C)** Gene set enrichment analysis of differentially-expressed genes between IDH-Mut-associated peripheral NK cells versus IDH-WT-associated peripheral NK cells across (A) Hallmark gene sets, and (B) KEGG gene sets. Pathways with false discovery rate (FDR) < 0.25 are shown. (C) Gene expression (normalized counts) of representative differentially expressed between IDH-Mut and IDH-WT-associated peripheral NK cells along with healthy donor NK cells.


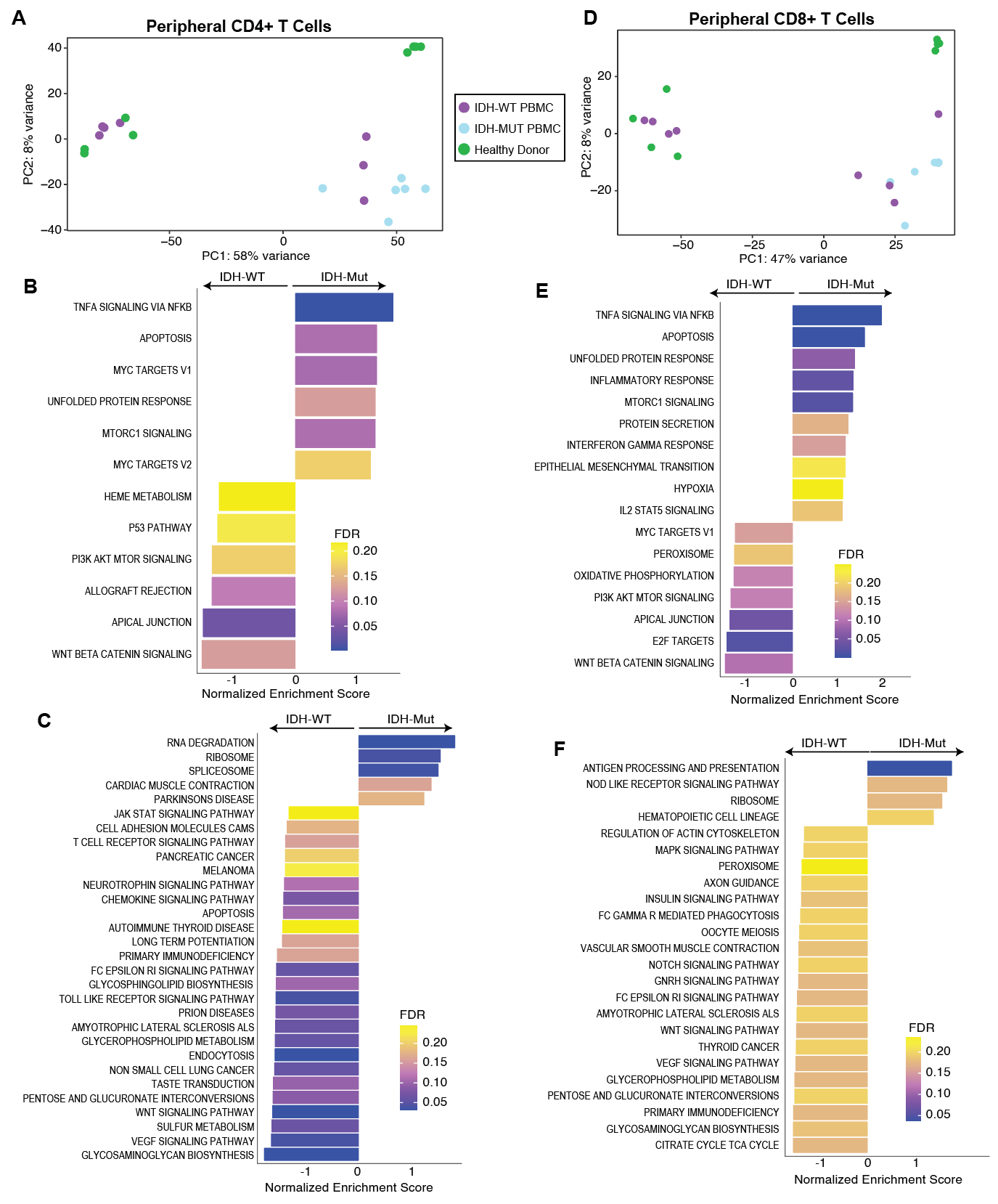


**Supplementary Figure 7. Pathway analysis of pseudobulk T cell populations. (A)** Principal component analysis (PCA) plot of pseudobulk RNA-seq profile of CD4+ T cells from PBMCs using all genes. Samples with at least 25 CD4+ cells in the PBMCs were included. **(B-C)** Gene set enrichment analysis of differentially-expressed genes between IDH-Mut-associated peripheral CD4+ T cells versus IDH-WT-associated peripheral CD4+ cells across (B) Hallmark gene sets, and (C) KEGG gene sets. Pathways with false discovery rate (FDR) < 0.25 are shown. Up to 30 top pathways are shown. **(D-F**) Pseudobulk and pathway analysis of CD8+ T cells showing (D) PCA plot, (E) Hallmark gene sets, and (F) KEGG gene sets as in (A-C).

**
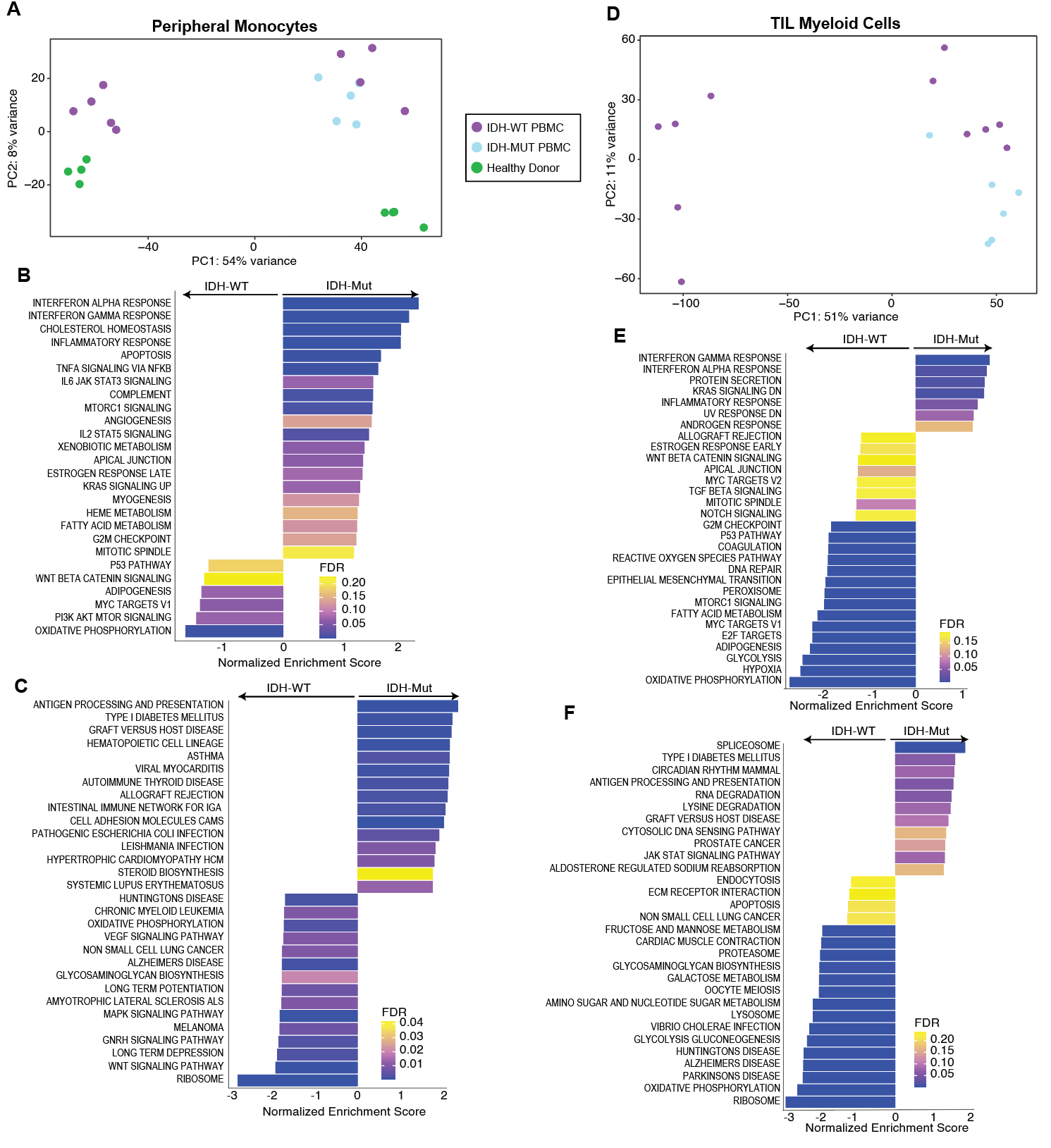
**

**Supplementary Figure 8. Pathway analysis of pseudobulk myeloid populations. (A)** Principal component analysis (PCA) plot of pseudobulk RNA-seq profile of monocytes from PBMCs using all genes. Samples with at least 25 monocytes in the PBMCs were included. **(B-C)** Gene set enrichment analysis of differentially-expressed genes between IDH-Mut-associated peripheral monocytes versus IDH-WT-associated peripheral monocytes across (B) Hallmark gene sets, and (C) KEGG gene sets. Pathways with false discovery rate (FDR) < 0.25 are shown. Up to 30 top pathways are shown. **(D-F**) Pseudobulk and pathway analysis of intratumoral macrophages + microglia showing (D) PCA plot, (E) Hallmark gene sets, and (F) KEGG gene sets as in (A-C).


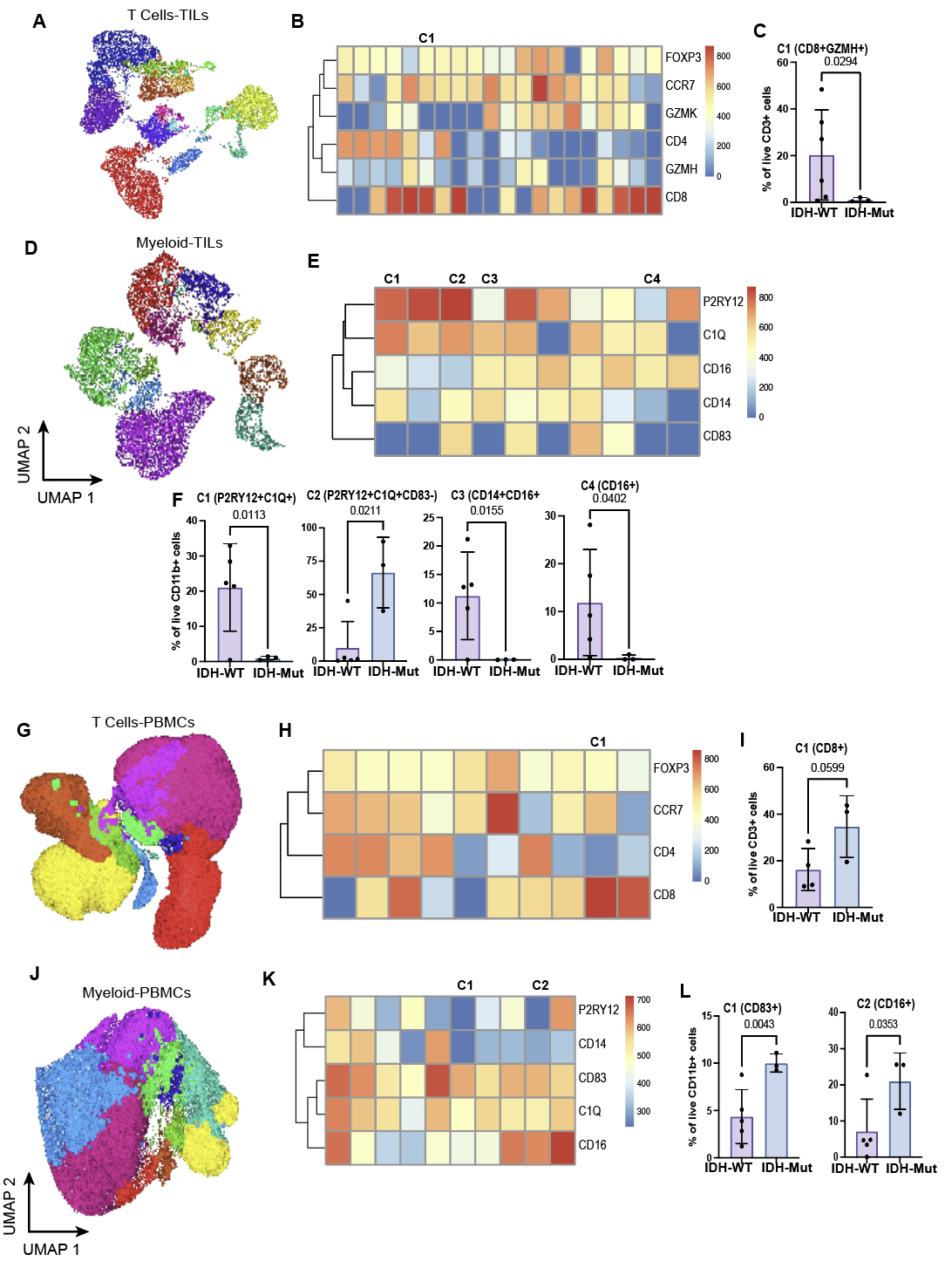


**Supplementary Figure 9. Flow cytometric analysis of TILs and PBMCs. (A-L)** For T-cells and myeloid cells in TILs and PBMCs, UMAP projections of 1500-25000 events are shown for IDH-Mut (n=3) and IDH-WT (n=5-6) samples. T-cells were gated manually for viable CD45^+^CD3^+^ cells and myeloid cells were gated for viable CD45^+^CD11b^+^ cells. Heatmaps show mean fluorescence intensity (MFI) of markers used in FlowSOM clustering. Cell type proportions from selected clusters are shown comparing IDH-Mut and IDH-WT samples.

**
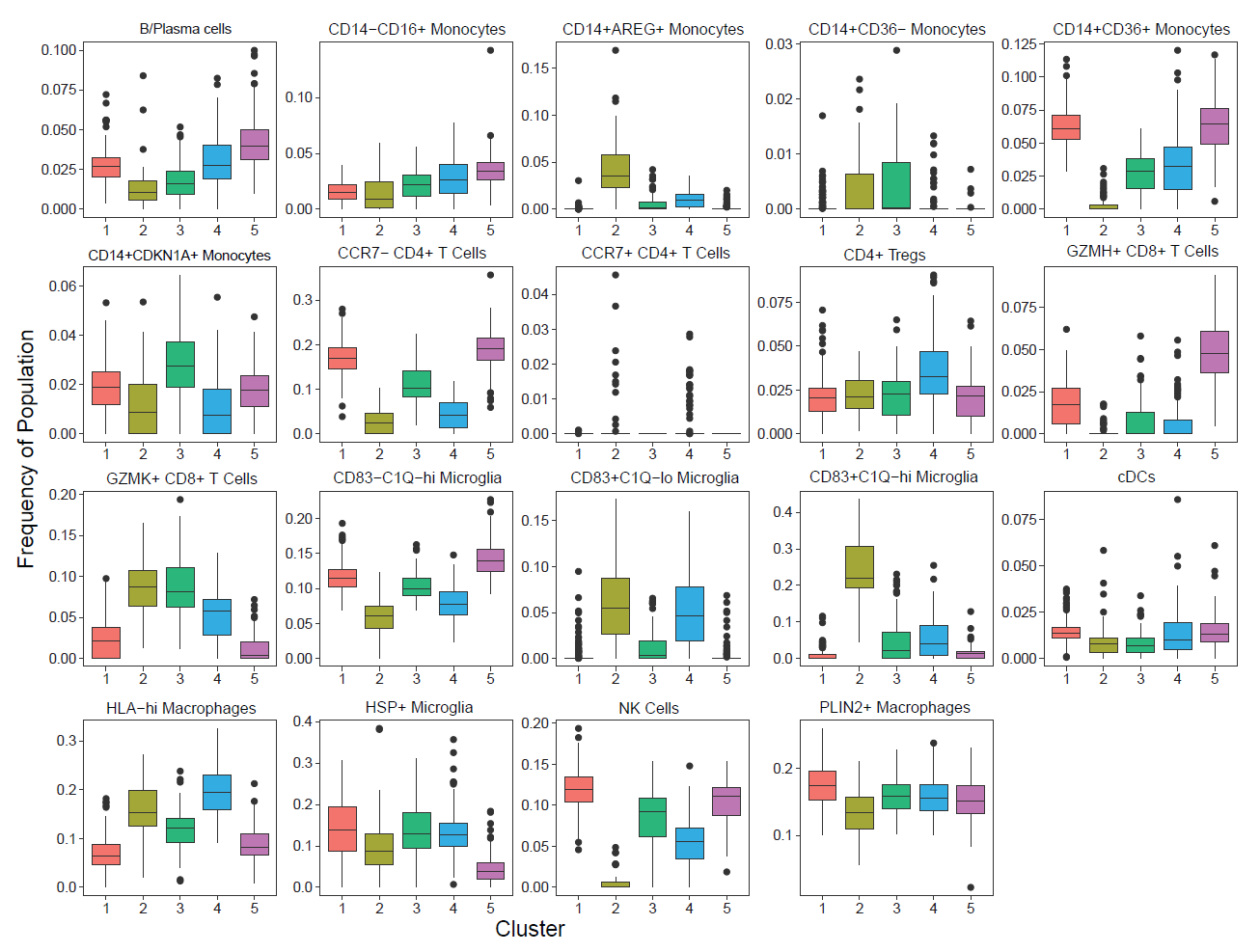
**

**Supplementary Figure 10.** Relative immune cell type composition across TCGA-GBM and TCGA-LGG cohort divided by immune-based clustering as defined in Figure 5B.

**
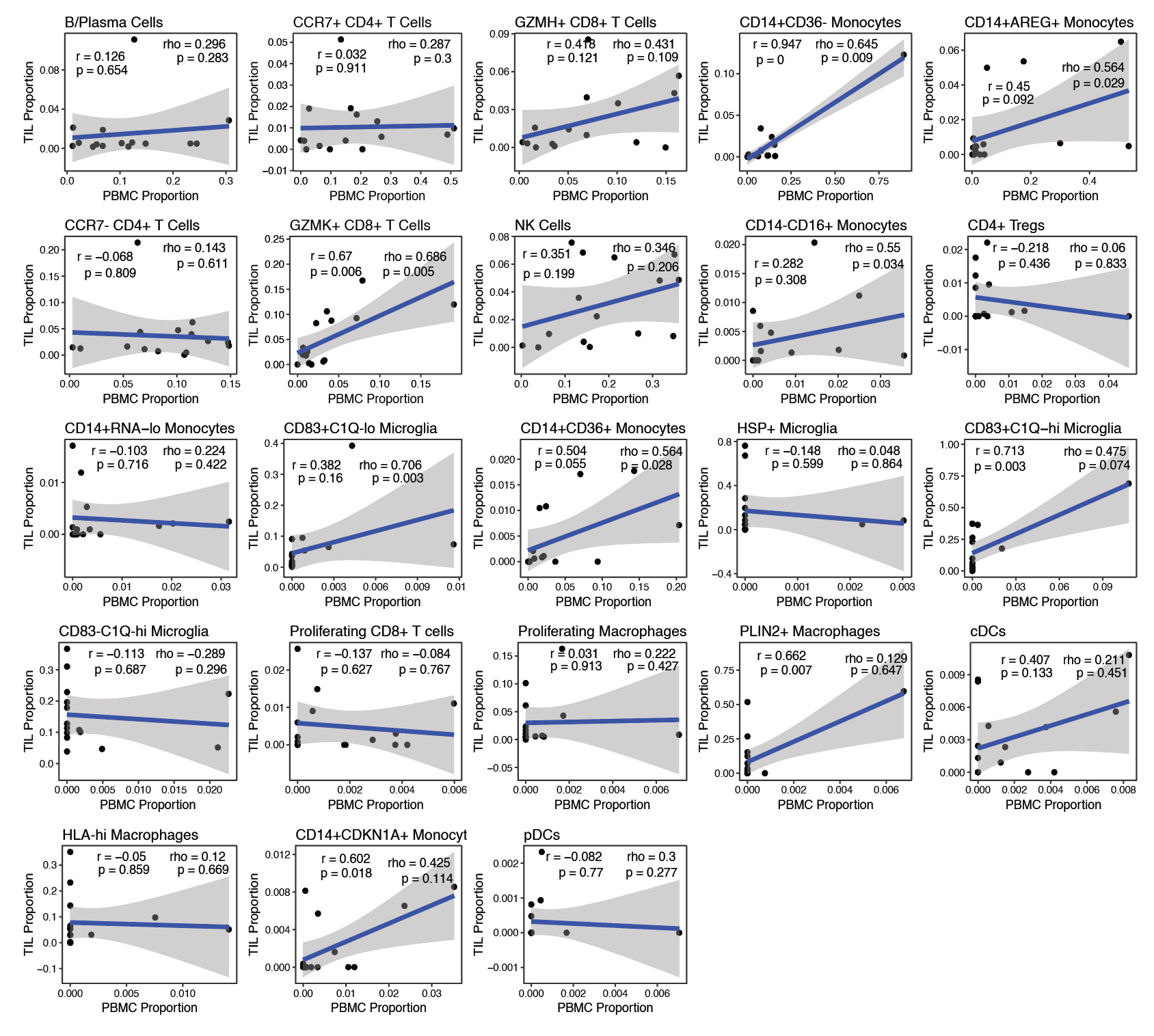
**

**Supplementary Figure 11.** Correlations between cell type proportions in PBMCs and TILs for each cell subpopulation as fraction of entire PBMC or TIL compartment for each patient*,* with Pearson’s correlation coefficient (*r*) and Spearman’s correlation coefficient (rho) along with respective p-values.

**
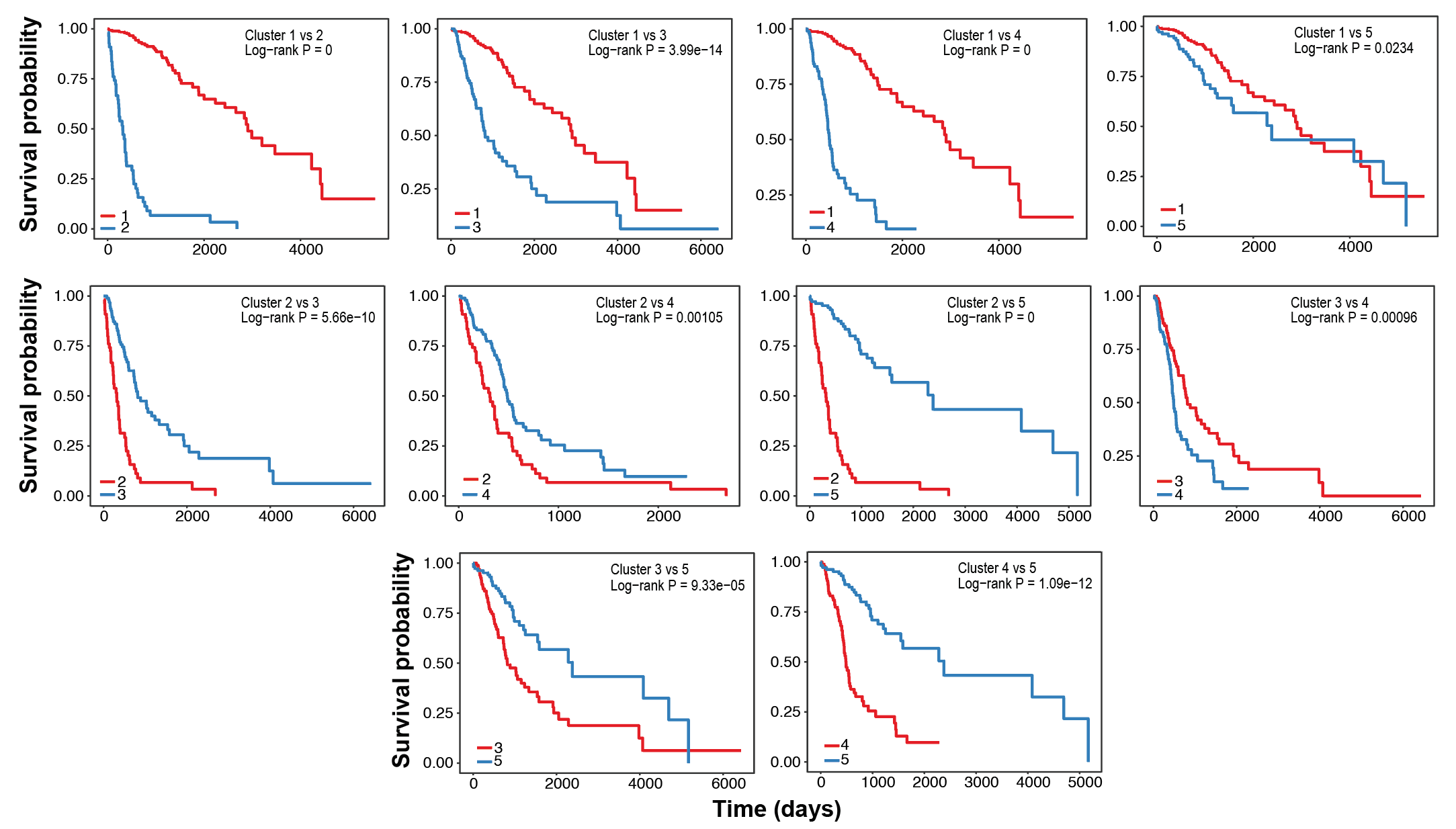
**

**Supplementary Figure 12.** Individual Kaplan-Meier plots for each pair of survival curves shown in Figure 4F. Significance was assessed using a log-rank test.

**
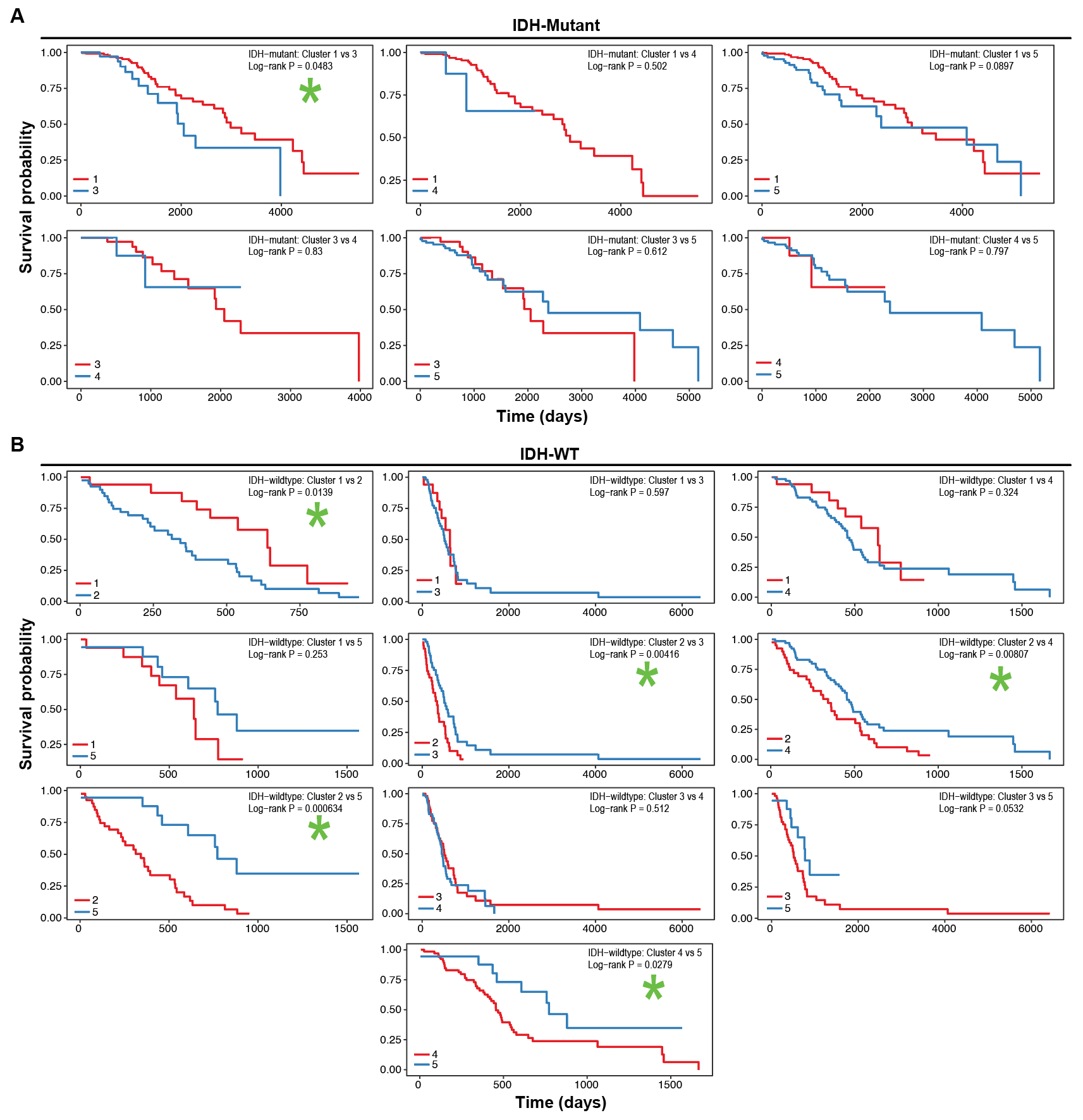
**

**Supplementary Figure 13.** Individual Kaplan-Meier plots for each pair of survival curves shown in Figure 4G, stratified by (A) IDH-Mut and (B) IDH-WT (B). Significance was assessed using a log-rank test. Pairs of curves with statistically significant differences in survival (p < 0.05) are highlighted with a green star.

**
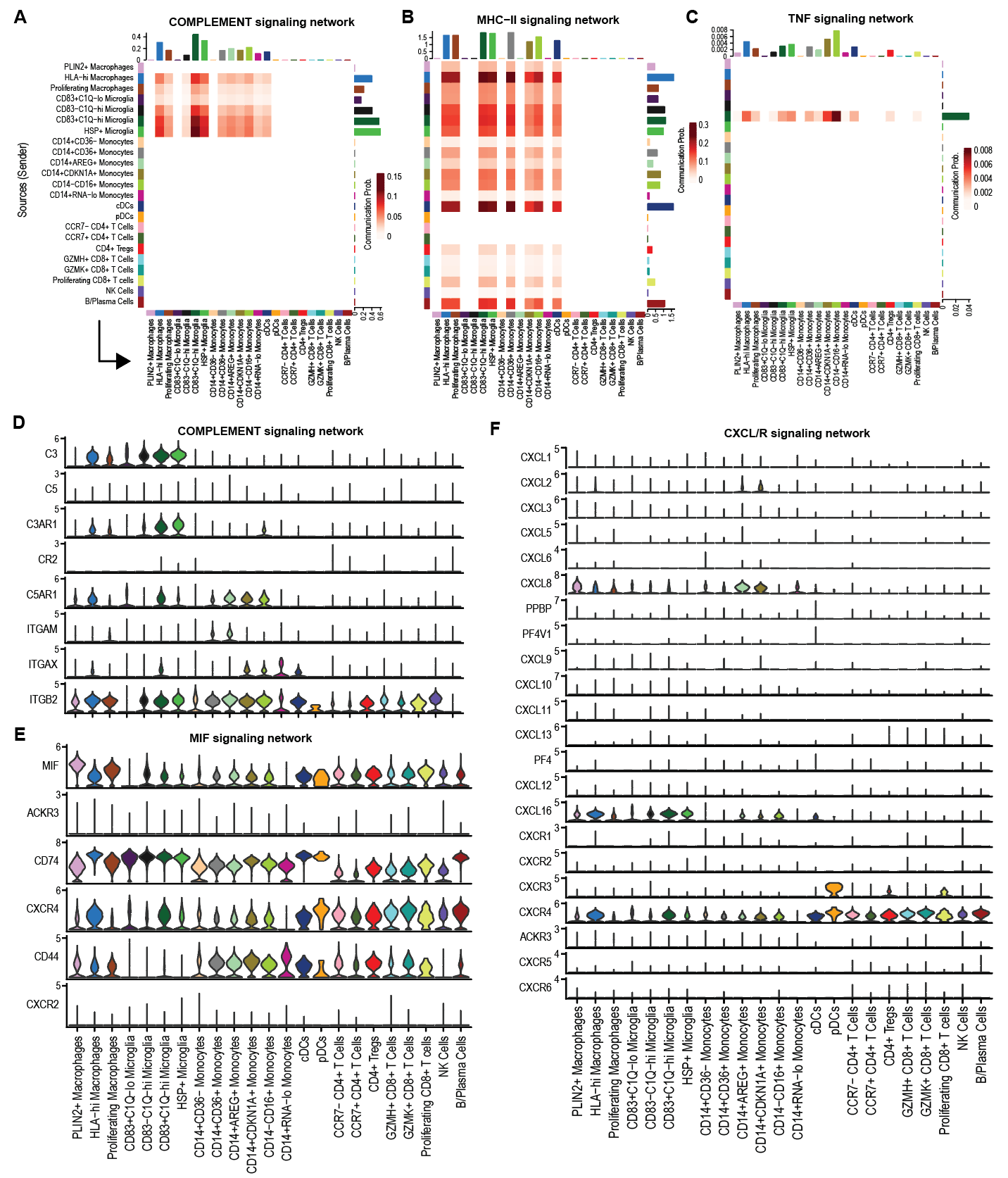
**

**Supplementary Figure 14. Ligand-receptor communication and gene expression of selected intercellular signaling pathways. (A-C)** Heatmap of predicted signaling via (A) complement pathway, (B) antigen-presentation pathway, (C) TNF pathway between all cell types. Color represents strength of signaling between two populations. The top bar plot represents the sum of the column values, and the right bar plot represents the sum of the row values. **(D-F)** Violin plots showing normalized gene expression of ligand and receptor genes present within the (D) complement, (E) MIF, and (F) CXCL/R signaling networks for each cell type.


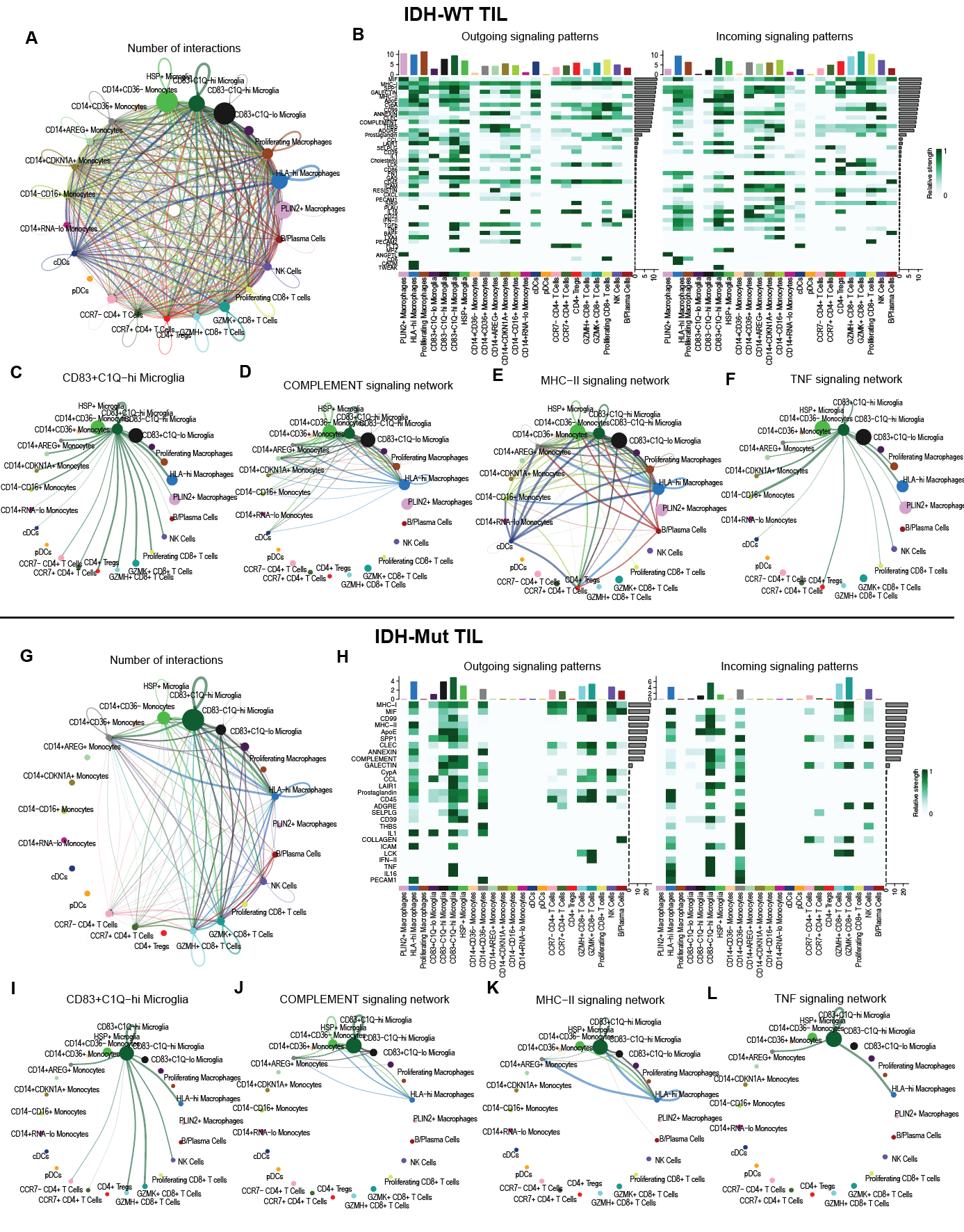


**Supplementary Figure 15. (A)** Aggregated cell-cell communication network as inferred by CellChat. Dot size indicates the relative number of cells of each cell type, and line thickness connecting cell types indicates the number of interactions between each cell type pair. Only IDH-WT TILs were included in the analysis. **(B)** Significant outgoing (ligand enriched) and incoming (receptor enriched) signaling predictions were scored for each annotated signaling pathway, and the strength of signaling (edge weights) for each cell type was scaled and plotted by pathway. The top barplot shows the total signaling strength of a cell group by summarizing all signaling pathways. The right barplot shows the total signaling strength (network edge weights for that group of ligand-receptor pairs) across all cell groups, illustrating whether a pathway is broadly activated. **(C)** Signaling sent from *CD83^+^C1Q^hi^* microglia. Dot size indicates relative cell abundance as in (A), and line thickness connecting cell types represents the total interaction strength (edge weights) between that cell type pair. **(D-F)** Network diagram of inferred signaling for (D) Complement, (E) MHC-II, and (F) TNF signaling pathways. Dot size indicates relative cell abundance as in (A), and line thickness represents the strength of the interaction aggregating edge weights for that group of ligand-receptor pairs. **(G-L)** Cell-cell communication network, pathway enrichment, and individual signaling networks as in (A-F). Only IDH-Mut TILs were included in the analysis.


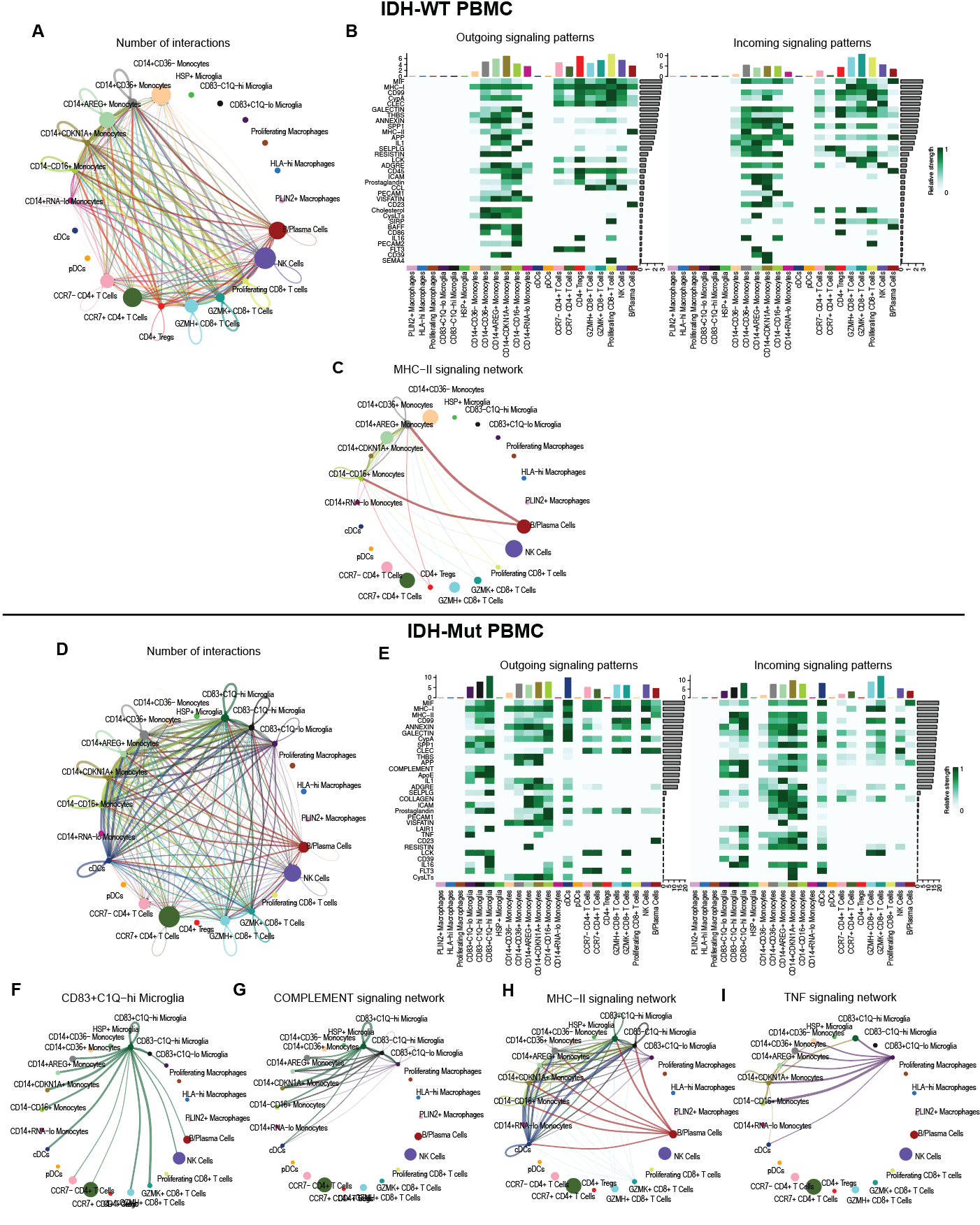


**Supplementary Figure 16. (A)** Aggregated cell-cell communication network as inferred by CellChat. Dot size indicates the relative number of cells of each cell type, and line thickness connecting cell types indicates the number of interactions between each cell type pair. Only IDH-WT PBMCs were included in the analysis. **(B)** Significant outgoing (ligand enriched) and incoming (receptor enriched) signaling predictions were scored for each annotated signaling pathway, and the strength of signaling (edge weights) for each cell type was scaled and plotted by pathway. The top barplot shows the total signaling strength of a cell group by summarizing all signaling pathways. The right barplot shows the total signaling strength (network edge weights for that group of ligand-receptor pairs) across all cell groups, illustrating whether a pathway is broadly activated. **(D)** Network diagram of inferred signaling for the MHC-II pathway. Dot size indicates relative cell abundance as in (A), and line thickness represents the strength of the interaction aggregating edge weights for that group of ligand-receptor pairs. There were no significant Complement and TNF signaling activity in IDH-WT PBMCs. **(D-E)** Cell-cell communication network and pathway enrichment, and individual signaling networks as in (A-B). Only IDH-Mut PBMCs were included in the analysis. **(F)** Signaling sent from *CD83^+^C1Q^hi^* microglia. Dot size indicates relative cell abundance as in (A), and line thickness connecting cell types represents the total interaction strength (edge weights) between that cell type pair. **(G-I)** Network diagram of inferred signaling for (G) Complement, (H) MHC-II, and (I) TNF signaling pathways. Dot size indicates relative cell abundance as in (D), and line thickness represents the strength of the interaction aggregating edge weights for that group of ligand-receptor pairs.

**Supplemental Methods**

**Patient cohort and sample acquisition for scRNA-seq**

Newly diagnosed glioma patients in whom at least 70% extent of resection could be achieved (as determined by the attend neurosurgical oncologist) were included in the study. On average, ~4g of grossly viable non-necrotic tissue were utilized for downstream analyses following confirmation by the attending pathologist. This paradigm ensured broad representation of tumor regions. Mutations were called using the CLIA-approved GlioSeq amplification-based targeted next-generation sequencing assay, as previously described^1^. This panel is capable of capturing 193 distinct genomic alterations, including mutations/indels/substitution (47 genes), chromosomal-level loss of heterozygosity (43 events), and gene fusions (103 events).

**Peripheral blood processing for scRNA-seq**

Peripheral blood was collected into 10mL vacutainer EDTA tubes (BD) at the time of surgery. Peripheral blood mononuclear cells (PBMCs) were isolated from whole blood by density gradient centrifugation. Briefly, whole blood was diluted 1:1 with Hanks Buffered Saline Solution (Thermo Fisher), and 30 mL of the diluted blood was layered over 15 mL of Ficoll-Hypaque (Fisher Scientific). Samples were then centrifuged at 400 x g for 20 minutes with the brake set to off. PBMCs were isolated and were washed with complete RPMI twice. PBMCs were then counted and either sorted for downstream scRNA-seq library generation or were cryopreserved for future analyses.

**Tissue processing for scRNA-seq**

Tissue obtained from the operating room was processed within 1 hour after resection. To process tumor tissues into a single-cell suspension, tissue was first manually mechanically disrupted into 1 cm^2^ fragments using a safety scalpel. These fragments were then resuspended in 5 mL of RPMI (Thermo Fisher) plus 50 ng/mL Liberase (Dispase Low) and cultured for 15 minutes at 37 ^o^C and 5% CO_2_. Following incubation, cells and tissue fragments in solution were passed over a 100 µm filter and washed with complete RPMI followed by centrifugation at 400 x g for 5 minutes. In addition to glioma tissue, normal cortex samples were obtained from tumor adjacent tissue (en route to the dissection). Isolated cells were then counted and either sorted for downstream scRNA-seq library generated or were cryopreserved for future analyses.

**Fluorescence activated cell sorting**

Single cell suspensions of PBMCs and tumor tissue were next sorted to remove debris and to isolate immune cells. PBMCs were sorted to ensure the cells were subjected to the same experimental conditions as tumor samples prior to scRNA-seq sample preparation. Cells were then stained for sorting by first incubating cells in a 1:4000 dilution of eFluour780 viability dye (eBioscience; Catalog number: 65-0863-14) in phosphate buffered saline (PBS) for 15 minutes at 4^o^C and were then washed and centrifuged at 400 x g for 5 minutes. Next, cells were incubated with a 1:100 dilution of PE conjugated monoclonal mouse anti-human CD45 (Biolegend, Catalog number: 368510) for 15 minutes at 4^o^C and were once again washed and spun down at 400 x g for 5 minutes. Cells were then resuspended in PBS supplemented with 2% FBS and live CD45+ cells were sorted form blood and tumor tissues. Sorting was performed using either a MoFlo Astrios or a Sony MA900 in the UPMC Hillman Cancer Center Flow Cytometry Core.

**Single-cell RNA-seq library preparation**

Following sorting, live CD45+ cells from blood and tumor tissue were centrifuged at 400 x g for 5 minutes. Cells were then resuspended in PBS with 0.04% bovine serum albumin and counted. Cells were then processed through the Chromium Controller (10x Genomics), targeting a recovery of 2,000 cells for individual samples or 4,000 cells for two cell hashed samples. Chromium Single Cell 3’ Reagent Kit v2 or the Chromium Single Cell 5’ Reagent Kit v1 (10x Genomics) were used to generate libraries according to the manufacturer’s instructions. Following droplet generation, reverse transcription was performed. Then, cDNA was amplified, and libraries were constructed with i7 sequencing adapters as per the manufacturer’s protocol.

**Next-generation sequencing**

Once final libraries were prepared, library concentration and size distributions were quantified using the TapeStation platform. If samples were found to have a significant quantity of free adapters, they were re-purified by SPRI selection and re-quantified. Samples were subsequently diluted, pooled, and sequenced on a NovaSeq6000 high-output kit at the UPMC Genome Center using sequencing parameters 28:8:0:90 (read1:i5:i7:read2, bp) targeting a sequencing depth of 50,000 – 80,000 reads/cell. Multiple samples were pooled and sequenced across 7 batches.

**Demultiplexing and genomic alignment**

After sequencing, unprocessed runs were downloaded from the UPMC Genome Center to the University of Pittsburgh High Throughput Computer Cluster. Bcl2fastq (v2.20.0) was used to demultiplex runs into individual samples. Following demultiplexing, reads were aligned to GRCh38 using Cell Ranger (10X Genomics v6.1.2), resulting in the creation of feature/barcode matrices for downstream analyses.

**Single-cell RNA-seq processing and integration**

Feature/barcode matrices output from Cell Ranger were read into the R package Seurat (v4.0.5)^2^ for downstream analyses. Healthy donor peripheral blood samples were obtained from previously published studies^3,4^. Data integration was performed using the reciprocal principal component analysis (RPCA) integration workflow as described. Briefly, samples were split based on chemistry (i.e., either 5’ or 3’ 10X Genomics reagents), and then we separately identified the top 2000 highly variable genes using the variance stabilizing transformation for each chemistry. Next, the sets of highly variable genes were separately scaled using the *ScaleData* function and a principal component analysis (PCA) was computed for each chemistry subset using the *RunPCA* function. We then identified anchors across the datasets using the first 30 principal components (PCs) and integrated the data using the *IntegrateData* function with default parameters.

**Single-cell RNA-seq annotation and differential gene analysis**

Using Seurat (v4.3), cells were clustered using the *FindNeighbors* function with the top 15 PCs followed by *FindClusters* and visualized using Uniform Manifold Approximation and Projection (UMAP) with the same principal components using the *RunUMAP* function. An initial annotation was performed using SingleR^5^ along with known marker genes. Two distinct clusters of neuroglial cells (astrocytes/neoplastic cells and oligodendrocytes) were identified expressing genes including *GFAP, MOG, S100B, OLIG2*, and *SOX2*, and without expression of any canonical immune cell markers. In total, 2,339 cells were filtered as contaminant populations, amounting to 4.3% of the processed data, leaving 51,880 cells in the final dataset. These clusters were excluded, and the data was re-clustered using *FindNeighbors* and *FindClusters*, and UMAP was recomputed for visualization using the top 15 PCs as above. Coarse annotations were based on the SingleR predictions and marker genes (**Supplementary Figure 1D**). Subsequently, myeloid cells and lymphoid cells were subset, re-clustered, and visualized by re-computing a UMAP projection. The final clustering resolutions were determined empirically. Differentially expressed genes were identified using *FindAllMarkers* function in Seurat using the Wilcoxon rank sum test with default parameters, and finer annotations were assigned based on these differentially expressed genes. Previously published macrophage/microglia gene signatures were applied to the data using the *AddModuleScore* function in Seurat. The initial coarse annotations were then revised based on the final sub-clustered annotations. DESeq2 was used for pseudobulk analysis and fgsea (v1.24.0) was used for pathway analysis with pathways from the Molecular Signatures Database^6^.

**SCENIC transcriptional regulatory network (TRN) analysis**

The Single-Cell Regulatory Network Inference and Clustering (SCENIC)^7^ was run using the pySCENIC implementation^8^ as previously described on the scRNA-seq data, after conversion of the Seurat object to loom format. The GRNBoost2 algorithm was used for GRN inference. To predict transcription factor regulons, we used the human v9 cisTarget motif collection and the hg38_refseq-r80 databases with the 500bpUp100Dw and TSS+/-10kb search spaces. All relevant databases were obtained from: https://resources.aertslab.org/cistarget/. The pySCENIC implementation of AUCell was used to score the activity of regulons for each cell. The *SCopeLoomR* package was then used to extract the regulons and AUCell matrix from the resulting loom file, and the final AUC matrix was added to the initial Seurat object for visualization and analysis. Differential regulons were identified using the *FindAllMarkers* function using the Wilcoxon rank sum test with the “*logfc.threshold* = 0.01.”

**Cellular interaction analysis**

CellChat^9^ was used for cellular interaction analysis using the CellChatDB database. All cell subtypes were included, although pDCs were filtered due to their low abundance. The significant cell-cell interactions were determined using CellChat default parameters, with population size accounted for and “trimean” used for calculating mean gene expression. Pathway annotations were taken from the original manuscript. CellChat native functions were used for visualization including *netVisual_aggregate*, *netVisual_circle*, *netVisual_bubble*, *netAnalysis_signalingRole_scatter*, *netVisual_heatmap*, *netAnalysis_signalingRole_heatmap*, and *plotGeneExpression*. In the primary analysis, we included all cells (TILs, PBMCs, non-neoplastic cortex) as shown in Figure 1B with the finer annotations for the myeloid and lymphoid populations as derived in Figures 2 and 3 respectively. This includes patient data and healthy donor cells. In supplementary analyses, we subset the cells by IDH mutation status and TILs/PBMCs, which does not include healthy donor cells.

**RNA velocity analysis**

RNA velocity was leveraged for pseudotemporal analysis. First, velocyto (v0.17)^10^ was used to generate feature/barcode matrices of unspliced gene expression data using the output from the CellRanger analyses as input. Next, scanpy (v1.8.2)^11^ and scVelo (v0.2.4)^12^ were used to combine the spliced and unspliced feature barcode matrices and the moments were identified based on the top 30 principal components and 50 nearest neighbors from the top 2,000 most highly variable genes. RNA velocity was then identified using the dynamical mode from scVelo. Finally, pseudotime was constructed from the underlying RNA velocity results.

**Spectral flow cytometry of peripheral blood**

Patent peripheral blood was collected utilizing EDTA-treated BD Vacutainer tubes. Blood was processed as described above. Samples were suspended in FBS with 10% DMSO and cryopreserved until use. After thawing at 37°C, samples were washed twice in FACS buffer (2% FBS in DPBS) and separated by centrifugation at 330 x *g* for 5 minutes at 4 °C. The supernatant was carefully removed to discard debris and freezing media. Cells were transferred to 96-well V-bottom plates and incubated with 10 mL viability stain (LIVE/DEAD Fixable Aqua, Invitrogen, 1:60 dilution) for 15 minutes at room temperature. Cells were then washed with cold FACS buffer and incubated with 1mg/mL anti-FcR (BD Pharmigen, 553142) and extracellular antibodies (1:50 dilution) for 30 minutes at ambient temperature. Cells were then washed twice with cold FACS buffer. Cells were fixed with IC fixation buffer (Invitrogen, 00-8222-49) at 4°C for 30 minutes. Cells were subsequently washed with cold FACS buffer and permeabilized (Invitrogen, 00-8333-56) before staining with intracellular antibodies (1:50 dilution) for 30 minutes at ambient temperature. Cells were washed with cold permeabilization buffer, then subsequently with cold FACS buffer. Cells were resuspended in 1% PFA in FACS buffer, transferred to cytometry tubes and stored at 4°C, protected from light until data collection. All data were collected on a Cytek Aurora spectral flow cytometer. Subsequent downstream analysis was performed in FlowJo v10 (TreeStar Inc). Antibody clones and fluorophores are listed in **Supplementary Table 6**.

**FlowSOM analysis**

Events were gated manually for viable CD45^+^, CD3^+^/CD11b^+^ single cells. Events were then downsampled to near the maximum event count of the sample with the lowest total event count, per sample. The downsampled events were then concatenated and the constituent samples were manually gated. UMAP projections were generated from the concatenated events with the UMAP Plugin (v4.0.4; all settings left at default). FlowSOM meta-clusters (total number chosen iteratively) were generated with the FlowSOM algorithm (3.0.18; all settings left at deafult with the exception of number of meta-clusters) on the concatenated events and then applied to the constituent samples. Statistical analysis of proportions of constituent IDH-Mut and IDH-WT viable CD45^+^, CD3^+^/CD11b^+^ cells within FlowSOM meta-clusters was performed using a one-sided t-test in R.

**Tissue processing for culture of neurospheres and conditioned media generation**

Tissue obtained for neurosphere culture from the operating room was processed within 1 hour following resection. Resected tissue was maintained in Accutase solution (Sigma Aldrich, A6964) and manually disrupted into sub-1 cm^3^ fragments utilizing a scalpel blade. Fragments were then serially dissociated via a flamed Pasteur pipette. Single cell suspensions were then homogenized via syringe aspiration and filtered through a 100 µm cell strainer. The cell suspension was pelleted at 330 *g*, resuspended in eBioscience RBC Lysis Buffer (Invitrogen, 00-4300-54), and incubated for 5 minutes at ambient temperature. Cells were then washed in PBS and resuspended in complete culture media (DMEM/F12 medium enriched with 1% B-27O Supplement (Gibco, 12587-010), 1% N-2 Supplement (Gibco, 17502-048, 1% HyClone Penicillin-Streptomycin (Cytiva, SV30010), and 0.02% Normocin (InvivoGen, ant-nr-1) and plated in 25 cm^2^ flask with vented lids. Cultures were maintained at 37 °C and 5% CO_2_ and fed weekly. Cultures were split at >80% confluence and the conditioned supernatant was collected and stored at -80°C for downstream assay use.

**Conditioned media assay**

PBMCs isolated from healthy volunteers were counted and plated in 12-well, flat-bottom tissue culture plates at 1x10^5^ cells per well in 1 mL of complete RPMI-1640 medium. Cells were incubated at 37 °C and 5% CO_2_ for 30 minutes while shaking at 80 RPM. Cells were then treated with conditioned media collected from patient-derived neurospheres for 24 hours at 37 °C and 5% CO_2_ for 30 minutes while shaking at 80 RPM. Positive control samples were incubated with complete RPMI-1640 and DMEM/F12 media (v/v), and negative controls were maintained in RPMI-1640. Nonadherent cells were then collected, and wells were washed with PBS. Supernatant was collected and spun down at 400 *g* at 4°C. Cell pellets were then processed for spectral flow cytometry, as described above.

**Isolation of microglia-like cells from PBMCs**

PBMCs were isolated from peripheral blood of an IDH-mutant glioma patient using Ficoll-Paque PREMIUM (Cytiva Life Sciences) density gradient centrifugation. Microglia-like cells were sorted from PBMCs by fluorescence-activated cell sorting (FACS) based on microglial surface marker expression (CD45^mid^P2RY12^+^) to ensure high-purity populations. The antibodies used for sorting are: anti-CD45 (BioLegend Inc), anti-CD83 (BD Biosciences), and anti-P2RY12 (Biolegend Inc).

**NK cell co-culture with microglia-like cells and tumor cells**

NK-92 cells (ATCC CRL-2407) were maintained in RPMI 1640 culture medium (Gibco supplemented with 10% FBS (Gibco), penicillin (100 UI/ml) and streptomycin (0.1 µg/ml) (Gibco). For co-culture experiments, NK-92 cells were either maintained in this complete medium with IL-2 (PeproTech) and IL-15 (PeproTech) supplementation (20 ng/mL each), or in cytokine-free medium (identical base medium without added IL-2 or IL-15) to evaluate the impact of exogenous interleukins on NK cell activation. Microglia-like cells (1.5×10^4^) were co-cultured with 3×10^4^ NK-92 cells at a 1:2 ratio (microglia:NK) in 6-well plates for 24 and 48 hours under both culture conditions (with and without IL-2/IL-15). In parallel, NK-alone controls were maintained under identical both culture conditions without microglia.

After the initial incubation (with or without microglia-like cells), NK-92 cells were incubated with IDH-mutant glioma neurospheres (as described above) at an effector-to-target ratio of 10:1 for 2 hours. Following the tumor co-culture, NK cells were stained for markers to assess activation status. The antibodies used were anti-CD69 (BD Biosciences) and anti-CD107a (BD Biosciences). A Zombie Aqua (BioLegend Inc) viability dye was used to exclude non-viable cells. Stained samples were acquired on a Cytek Aurora flow cytometer and analyzed using FlowJo Software (BD Biosciences). NK-92 cells were identified based on CD45^+^CD56^+^ expression, with doublets and dead cells excluded from analysis.

**Analysis of glioma patients from TCGA**

RNA-seq gene expression counts, somatic mutation data, and clinical data were downloaded from the TCGA using the R package TCGABiolinks^13^. We sought to identify groups of patients based on somatic mutations status and the frequencies of immune cells through deconvolution as described below. To achieve this, we performed principal component analysis using the frequencies of immune cells across brain tumor patients from TCGA, including the TCGA-GBM and TCGA-LGG projects. We identified clusters of patients in PCA space using k-means clustering. We then looked for relationships between immune clusters and somatic mutations and patient outcomes. Finally, we used a cross-validation machine-learning based Cox-regression survival analysis to identify features that were associated with outcome. This analysis was implemented using the R package caret^14^.

**Deconvolution of TCGA data and survival analysis**

CIBERSORTx^15^ was employed to infer the frequency of different immune cell subsets across patients from the TCGA database. Briefly, a reference matrix was constructed based on the cell types identified in our single-cell RNA-seq data using the TIL data without downsampling. This reference matrix was then used to deconvolute the frequencies of immune cell subsets across patients from the TCGA. CIBERSORTx was run as a singularity instance on the Pitt Center for Research Computing High Throughput Cluster, with count data from our scRNA-seq dataset and brain tumor patients from the TCGA as input. S-mode was used for batch correction, and all other parameters were kept as defaults.

Survival analysis was performed using Cox proportional hazard’s regression in the R package survival (v3.5-3). In the Cox regression machine learning model, the following variables were included: immune clusters, mutation status for ATRX, CIC, EGFR, FLG, IDH1, LRP2, MUC16, MUC17, PCLO, PIK3CA, PKHD1, PTEN, RYR2, TP53, TTN, MGMT promoter status, and 1p/19q co-deletion status (**Supplementary Table 7**). We plotted the top variables with a relationship to survival and subsequently included those in the Cox model.

**Data and code availability**

Raw demultiplexed sequencing data and processed feature/barcode matrices are available on the Gene Expression Omnibus under accession number GSE247824. No new software was created for this study. Standard pipelines and analytical packages were employed as previously described.

Any additional information required to reanalyze the data reported in this paper is available from the lead contact upon request: Nduka Amankulor ([nduka.amankulor@pennmedicine.upenn.edu](mailto:nduka.amankulor@pennmedicine.upenn.edu)).

**Supplemental References**

1. Nikiforova MN, Wald AI, Melan MA, et al. Targeted next-generation sequencing panel (GlioSeq) provides comprehensive genetic profiling of central nervous system tumors. *Neuro-Oncol*. 2016;18(3):379-387. doi:10.1093/neuonc/nov289

2. Hao Y, Hao S, Andersen-Nissen E, et al. Integrated analysis of multimodal single-cell data. *Cell*. 2021;184(13):3573-3587.e29. doi:10.1016/j.cell.2021.04.048

3. Cillo AR, Kürten CH, Tabib T, et al. Immune landscape of viral- and carcinogen-driven head and neck cancer. *Immunity*. 2020;52(1):183-199.e9. doi:10.1016/j.immuni.2019.11.014

4. Cillo AR, Somasundaram A, Shan F, et al. People critically ill with COVID-19 exhibit peripheral immune profiles predictive of mortality and reflective of SARS-CoV-2 lung viral burden. *Cell Rep Med*. 2021;2(12):100476. doi:10.1016/j.xcrm.2021.100476

5. Aran D, Looney AP, Liu L, et al. Reference-based analysis of lung single-cell sequencing reveals a transitional profibrotic macrophage. *Nat Immunol*. 2019;20(2):163-172. doi:10.1038/s41590-018-0276-y

6. Liberzon A, Birger C, Thorvaldsdóttir H, Ghandi M, Mesirov JP, Tamayo P. The Molecular Signatures Database (MSigDB) hallmark gene set collection. *Cell Syst*. 2015;1(6):417-425. doi:10.1016/j.cels.2015.12.004

7. Aibar S, González-Blas CB, Moerman T, et al. SCENIC: single-cell regulatory network inference and clustering. *Nat Methods*. 2017;14(11):1083-1086. doi:10.1038/nmeth.4463

8. Van de Sande B, Flerin C, Davie K, et al. A scalable SCENIC workflow for single-cell gene regulatory network analysis. *Nat Protoc*. 2020;15(7):2247-2276. doi:10.1038/s41596-020-0336-2

9. Jin S, Guerrero-Juarez CF, Zhang L, et al. Inference and analysis of cell-cell communication using CellChat. *Nat Commun*. 2021;12(1):1088. doi:10.1038/s41467-021-21246-9

10. La Manno G, Soldatov R, Zeisel A, et al. RNA velocity of single cells. *Nature*. 2018;560(7719):494-498. doi:10.1038/s41586-018-0414-6

11. Wolf FA, Angerer P, Theis FJ. SCANPY: large-scale single-cell gene expression data analysis. *Genome Biol*. 2018;19:15. doi:10.1186/s13059-017-1382-0

12. Bergen V, Lange M, Peidli S, Wolf FA, Theis FJ. Generalizing RNA velocity to transient cell states through dynamical modeling. *Nat Biotechnol*. 2020;38(12):1408-1414. doi:10.1038/s41587-020-0591-3

13. Colaprico A, Silva TC, Olsen C, et al. TCGAbiolinks: an R/Bioconductor package for integrative analysis of TCGA data. *Nucleic Acids Res*. 2016;44(8):e71. doi:10.1093/nar/gkv1507

14. Kuhn M. Building Predictive Models in R Using the caret Package. *J Stat Softw*. 2008;28:1-26. doi:10.18637/jss.v028.i05

15. Newman AM, Steen CB, Liu CL, et al. Determining cell type abundance and expression from bulk tissues with digital cytometry. *Nat Biotechnol*. 2019;37(7):773-782. doi:10.1038/s41587-019-0114-2
